# Supplementary material for: Ribo-ODDR: oligo design pipeline for experiment-specific rRNA depletion in Ribo-seq
Source: Bioinformatics. 2021 Mar 15;37(17):2659–67. doi: 10.1093/bioinformatics/btab171 (PMC8428583; doi:10.1093/bioinformatics/btab171)
Supplement: btab171_Supplementary_Data [file btab171_supplementary_data.pdf]

Ribo-ODDR: *O*ligo *D*esign pipeline for experiment-specific rRNA *D*epletion in *R*ibo-seq
(Supplementary Document)

Ferhat Alkan [†,*], Joana Silva [†], Eric Pintó Barberà, William J Faller [*]

Division of Oncogenomics, Netherlands Cancer Institute, 1066 CX, Amsterdam, The Netherlands

[†] Joint first authors
[*] Corresponding Author, email: w.faller@nki.nl;
software-related correspondence can be addressed to f.alkan@nki.nl

List of Figures

Supplementary Figure 1 Screenshot from the oligo selection user interface, *Ribo-ODDR oligo-selector*. . . 3
Supplementary Figure 2 Analysis of an additional mouse Ribo-seq dataset generated using Ribo-Zero. . . 3
Supplementary Figure 3 Suboptimal performance of RiboCop rRNA depletion kit in a Ribo-seq experiment using *in vitro* mouse intestinal organoids. 4
Supplementary Figure 4 Sample-specificity of rRNA fragments and cross-replicate correlation analysis of oligo depleting potentials for brain 4
Supplementary Figure 5 Sample-specificity of rRNA fragments and cross-replicate correlation analysis of oligo depleting potentials for heart 4
Supplementary Figure 6 Sample-specificity of rRNA fragments and cross-replicate correlation analysis of oligo depleting potentials for kidney 5
Supplementary Figure 7 Sample-specificity of rRNA fragments and cross-replicate correlation analysis of oligo depleting potentials for liver 5
Supplementary Figure 8 Sample-specificity of rRNA fragments and cross-replicate correlation analysis of oligo depleting potentials for skeletal 5
Supplementary Figure 9 Sample-specificity of rRNA fragments and cross-replicate correlation analysis of oligo depleting potentials for testis 6
Supplementary Figure 10 Sample-specificity of rRNA fragments and cross-replicate correlation analysis of oligo depleting potentials for lung 6
Supplementary Figure 11 Sample-specificity of rRNA fragments and cross-replicate correlation analysis of oligo depleting potentials for pancreas 7
Supplementary Figure 12 Sample-specificity of rRNA fragments and cross-replicate correlation analysis of oligo depleting potentials for spleen 7
Supplementary Figure 13 Tissue and RNase specificity of rRNA fragments in mouse Ribo-seq experiments. 8
Supplementary Figure 14 RNase specificity of rRNA fragments in Ribo-seq experiments with mouse liver. 8
Supplementary Figure 15 Ribo-seq produce different rRNA fragments in mouse liver samples with different oncogenic driver mutations. 9
Supplementary Figure 16 rRNA abundance profiles of *in vitro* Ribo-seq experiments with human (SET-0) and SET-1 oligos. 10
Supplementary Figure 17 Alternative visualisation for rRNA abundance profiles of *in vivo* Ribo-seq experiments with SET-1 and SET-2 oligos. 11
Supplementary Figure 18 Read count analysis for potential off-targets of new SET2 oligos. 12
Supplementary Figure 19 Quality Control plots for the Ribo-seq experiment mc1_w_SET1. 15

Supplementary Figure 20 Quality Control plots for the Ribo-seq experiment mc2_w_SET1. 16
Supplementary Figure 21 Quality Control plots for the Ribo-seq experiment mc3_w_SET1. 17
Supplementary Figure 22 Quality Control plots for the Ribo-seq experiment mc4_w_SET2. 18
Supplementary Figure 23 Quality Control plots for the Ribo-seq experiment mc5_w_SET2. 19
Supplementary Figure 24 Quality Control plots for the Ribo-seq experiment mc6_w_SET2. 20
Supplementary Figure 25 Quality Control plots for the Ribo-seq experiment mc7_w_SET3. 21
Supplementary Figure 26 Quality Control plots for the Ribo-seq experiment mc8_w_SET3. 22
Supplementary Figure 27 Quality Control plots for the Ribo-seq experiment mc9_RiboZero. 23
Supplementary Figure 28 Quality Control plots for the Ribo-seq experiment mc10_RiboZero. 24
Supplementary Figure 29 Quality Control plots for the Ribo-seq experiment mc11_RiboCop. 25
Supplementary Figure 30 Quality Control plots for the Ribo-seq experiment mc12_RiboCop. 26
Supplementary Figure 31 Quality Control plots for the Ribo-seq experiment org1_RiboCop. 27
Supplementary Figure 32 Quality Control plots for the Ribo-seq experiment org1_SET0. 28
Supplementary Figure 33 Quality Control plots for the Ribo-seq experiment org2_SET1. 29
Supplementary Figure 34 Quality Control plots for the Ribo-seq experiment org3_SET1. 30

List of Tables

Supplementary Table 1 Size selection marker, adapter and primer sequences. 13
Supplementary Table 2 Sequences for human oligos (SET-0). 13
Supplementary Table 3 Sequences for SET-1 oligos. 13
Supplementary Table 4 Sequences for SET-2 oligos. 14
Supplementary Table 5 Sequences for SET-3 oligos. 14

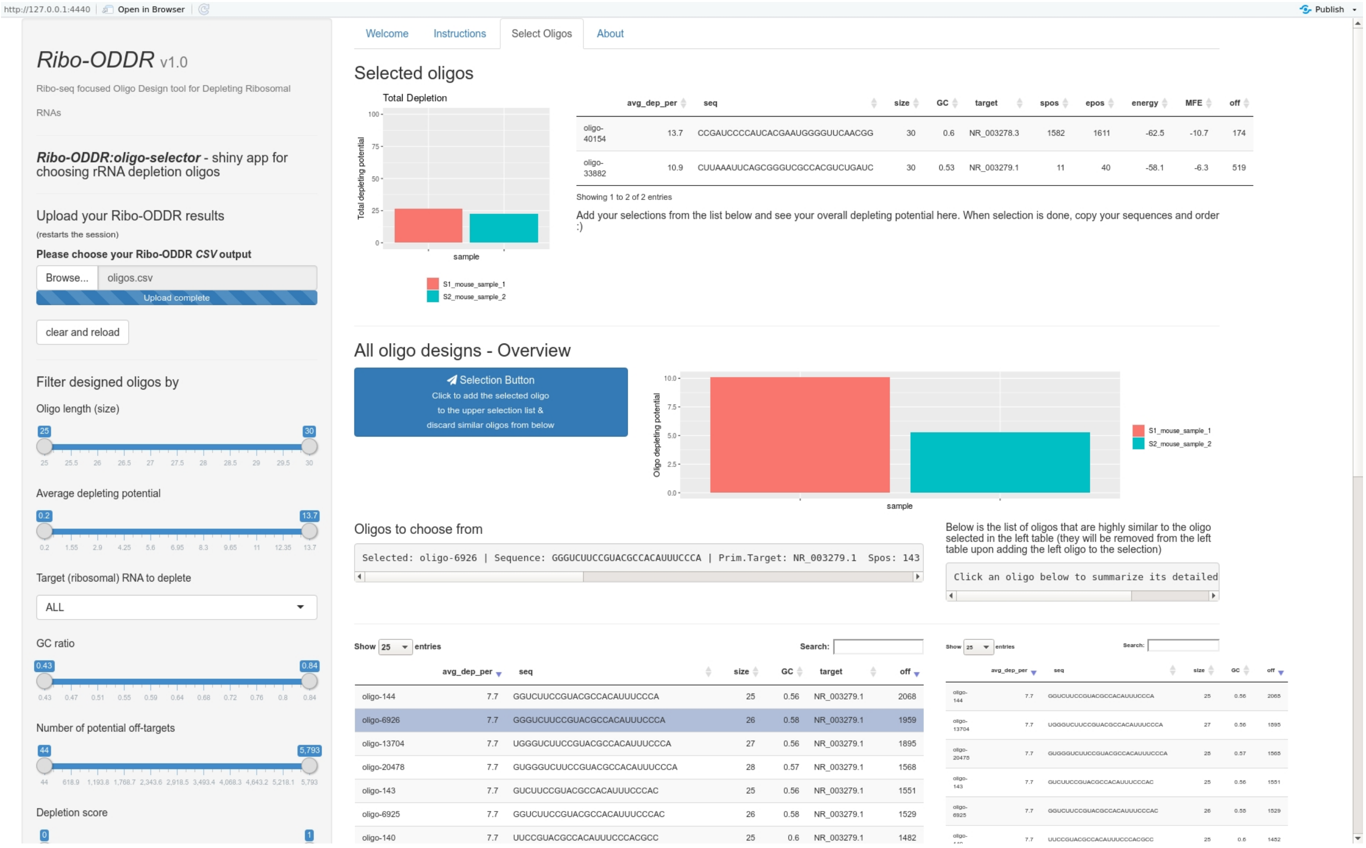

Supplementary Figure 1: Screenshot from the oligo selection user interface, *Ribo-ODDR oligo-selector*.

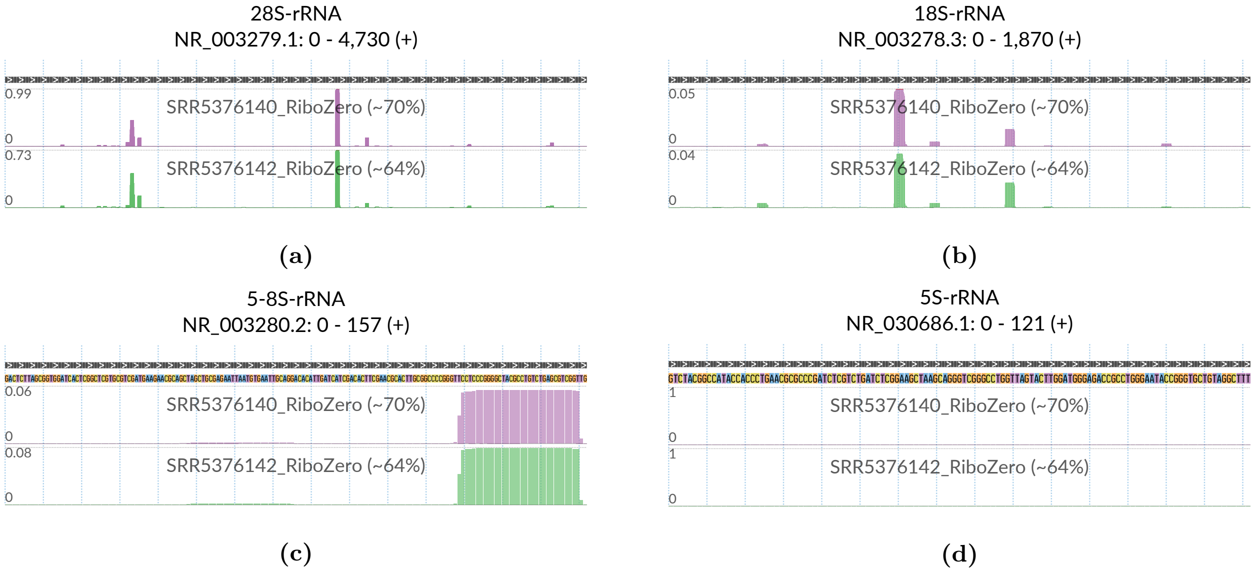

(a) (b) (c) (d)

Supplementary Figure 2: Suboptimal performance of Ribo-Zero rRNA depletion kit in Ribo-seq. Visualization is based on a public dataset[1] where Ribo-Zero kit was used for rRNA depletion. Each track shows the positional abundance profile of 28S (a), 18S (b), 5-8S (c) and 5S (d) rRNA fragments coming from individual samples. For every position in the x-axis, y-axis represents the normalized read ratio, number of rRNA reads mapped to that position divided by the total number of reads mapped to all protein coding transcripts. Sample-specific total rRNA percentages are given in track labels together with public SRA accession IDs for analyzed experiments.

[1] Simsek D, Tiu GC, Flynn RA, Byeon GW, Leppek K, Xu AF, Chang HY, Barna M. The Mammalian Ribo-interactome Reveals Ribosome Functional Diversity and Heterogeneity. Cell. 2017 Jun; 169(6):1051–1065.

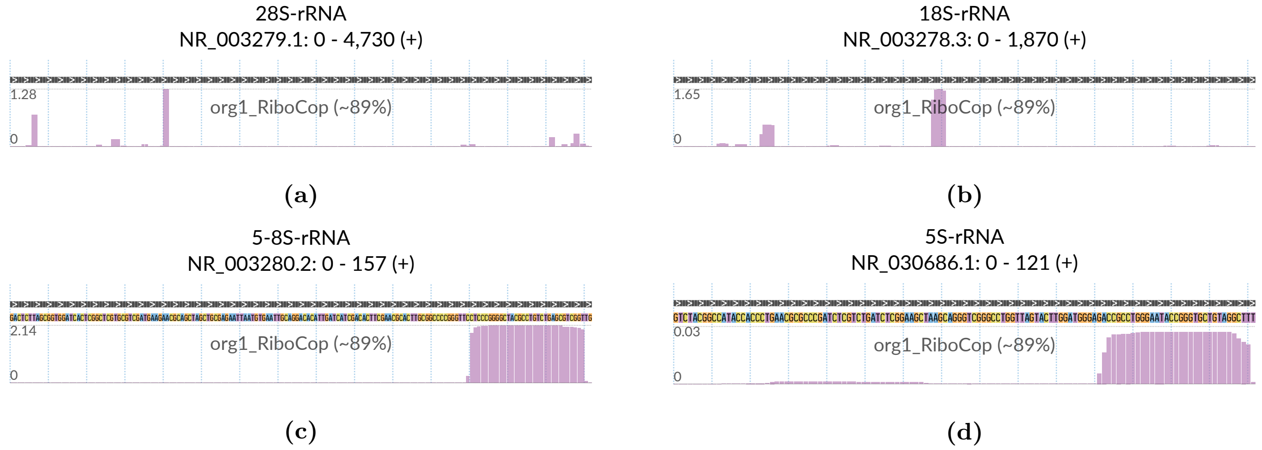

Supplementary Figure 3: Suboptimal performance of RiboCop rRNA depletion kit in Ribo-seq. Visualization is based on an *in vitro* Ribo-seq experiment using RiboCop, performed in mouse intestinal organoids. The track shows the positional abundance profile of 28S (a), 18S (b), 5-8S (c) and 5S (d) rRNA fragments. For every position in the x-axis, y-axis represents the normalized read ratio, number of rRNA reads mapped to that position divided by the total number of reads mapped to all protein coding transcripts. Total rRNA percentage is given in the track label.

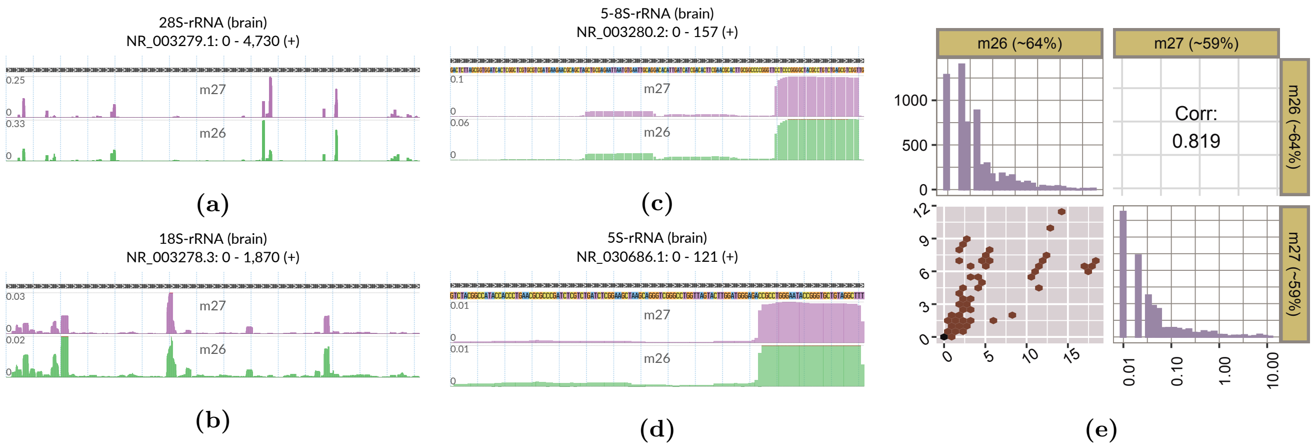

Supplementary Figure 4: Sample-specific rRNA fragment profiles (a-d) of **brain** replicates and the cross-replicate correlation analysis (e) of oligo depleting potentials computed by *Ribo-ODDR*. These figures are generated in a similar way to Figure 2&3 of the original paper, with the exception of being sample-specific over all **brain** replicates instead of being organ-specific at each row and column.

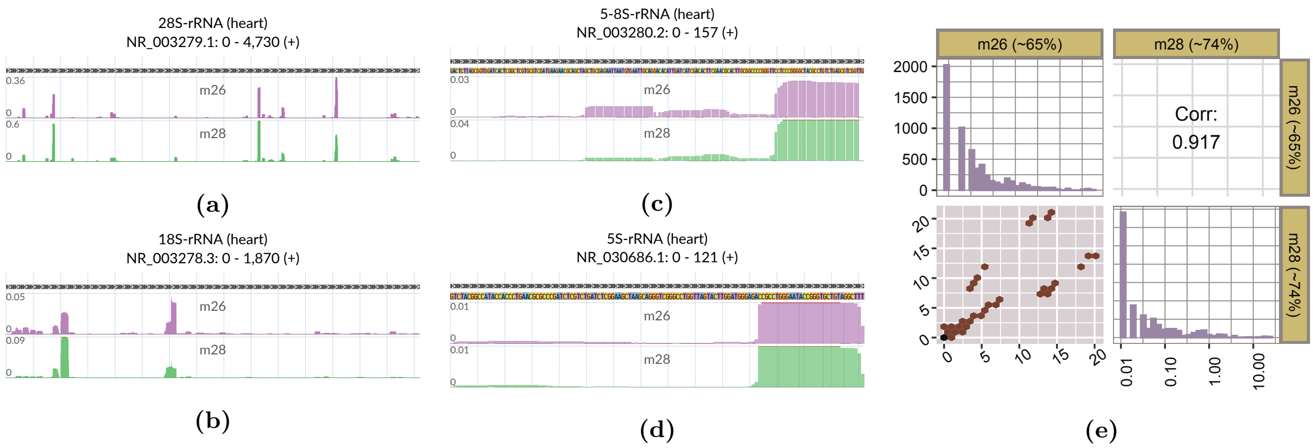

Supplementary Figure 5: Sample-specific rRNA fragment profiles (a-d) of **heart** replicates and the cross-replicate correlation analysis (e) of oligo depleting potentials computed by *Ribo-ODDR*. These figures are generated in a similar way to Figure 2&3 of the original paper, with the exception of being sample-specific over all **heart** replicates instead of being organ-specific at each row and column.

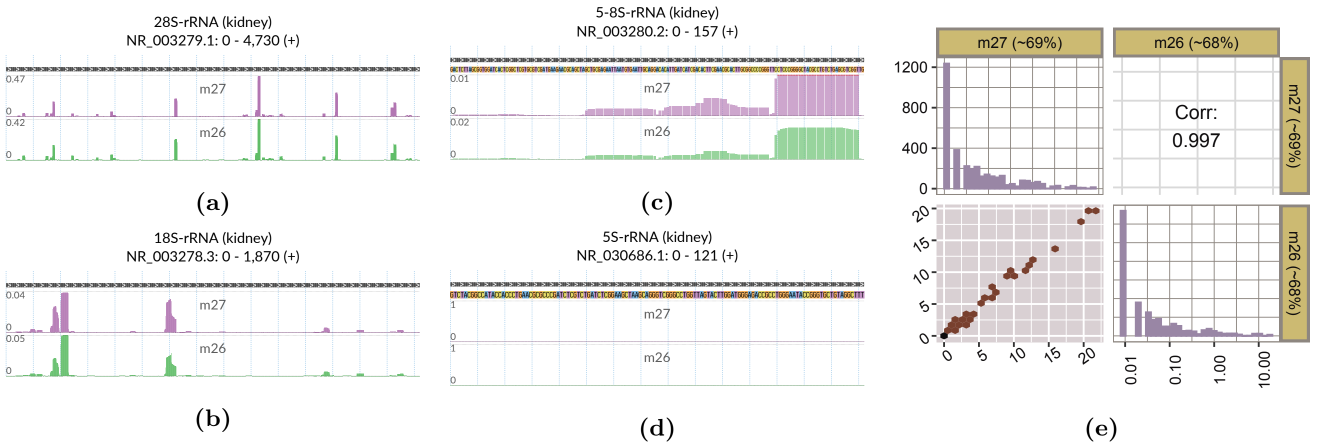

Supplementary Figure 6: Sample-specific rRNA fragment profiles (a-d) of **kidney** replicates and the cross-replicate correlation analysis (e) of oligo depleting potentials computed by *Ribo-ODDR*. These figures are generated in a similar way to Figure 2&3 of the original paper, with the exception of being sample-specific over all **kidney** replicates instead of being organ-specific at each row and column.

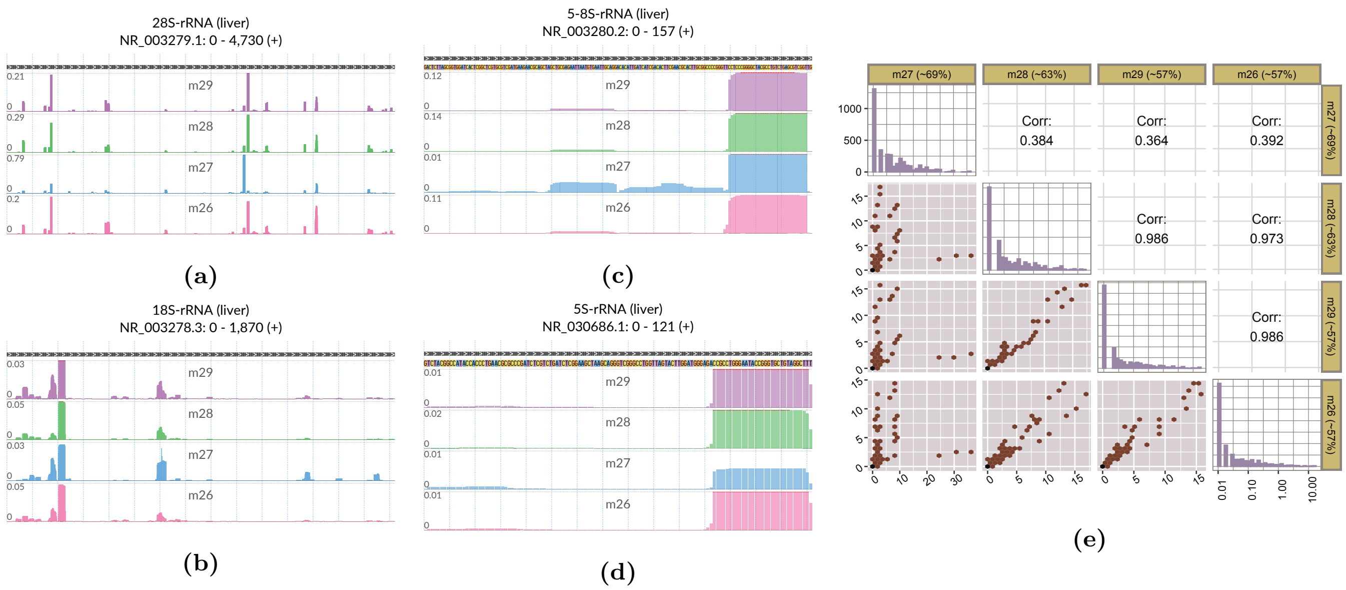

Supplementary Figure 7: Sample-specific rRNA fragment profiles (a-d) of **liver** replicates and the cross-replicate correlation analysis (e) of oligo depleting potentials computed by *Ribo-ODDR*. These figures are generated in a similar way to Figure 2&3 of the original paper, with the exception of being sample-specific over all **liver** replicates instead of being organ-specific at each row and column.

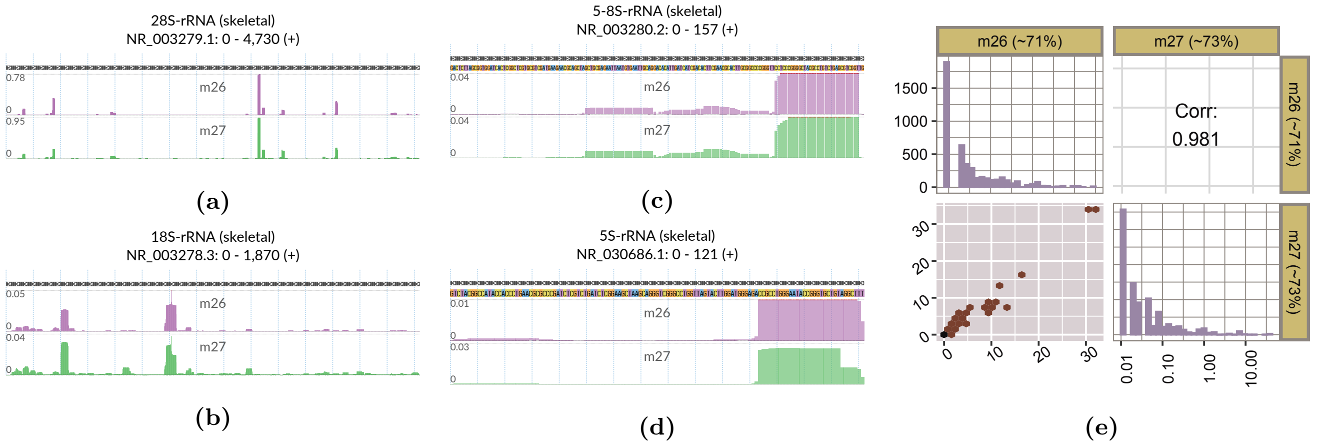

Supplementary Figure 8: Sample-specific rRNA fragment profiles (a-d) of **skeletal** replicates and the cross-replicate correlation analysis (e) of oligo depleting potentials computed by *Ribo-ODDR*. These figures are generated in a similar way to Figure 2&3 of the original paper, with the exception of being sample-specific over all **skeletal** replicates instead of being organ-specific at each row and column.

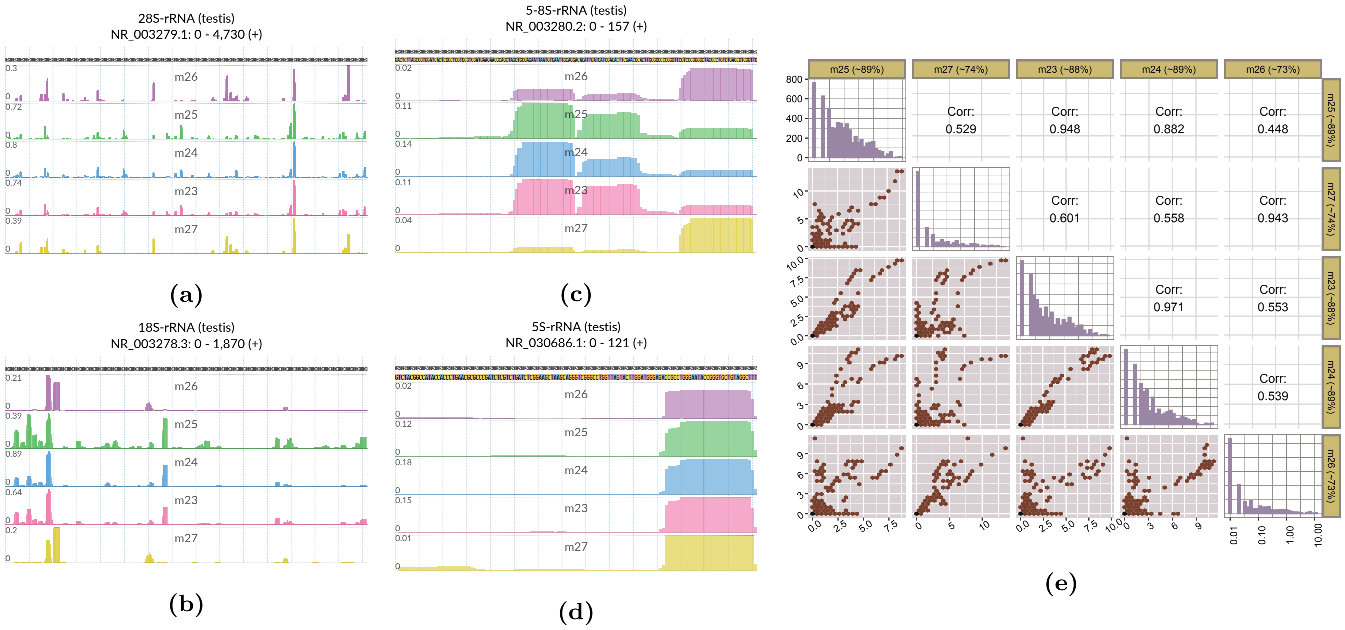

Supplementary Figure 9: Sample-specific rRNA fragment profiles (a-d) of **testis** replicates and the cross-replicate correlation analysis (e) of oligo depleting potentials computed by *Ribo-ODDR*. These figures are generated in a similar way to Figure 2&3 of the original paper, with the exception of being sample-specific over all **testis** replicates instead of being organ-specific at each row and column.

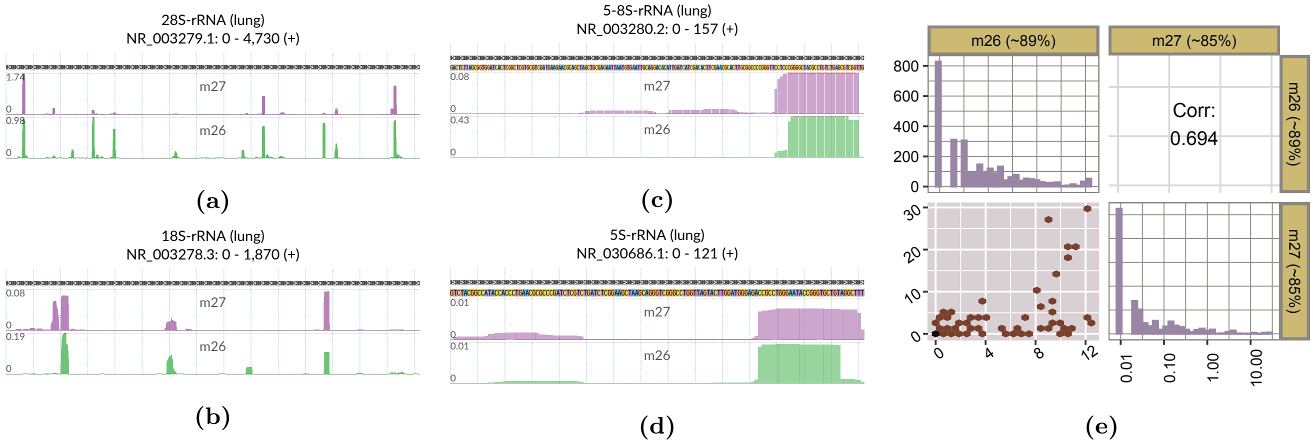

Supplementary Figure 10: Sample-specific rRNA fragment profiles (a-d) of **lung** replicates and the cross-replicate correlation analysis (e) of oligo depleting potentials computed by *Ribo-ODDR*. These figures are generated in a similar way to Figure 2&3 of the original paper, with the exception of being sample-specific over all **lung** replicates instead of being organ-specific at each row and column.

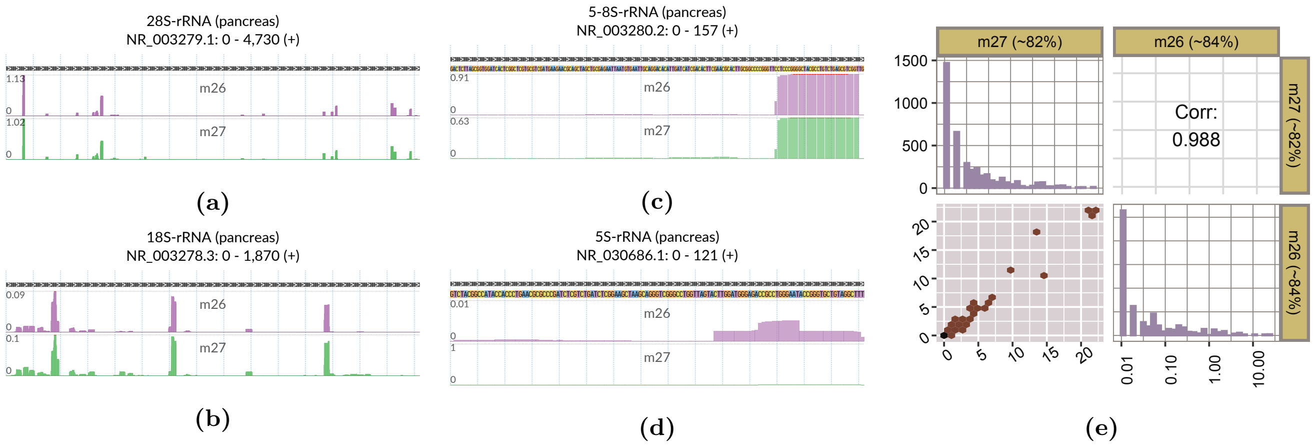

Supplementary Figure 11: Sample-specific rRNA fragment profiles (a-d) of **pancreas** replicates and the cross-replicate correlation analysis (e) of oligo depleting potentials computed by *Ribo-ODDR*. These figures are generated in a similar way to Figure 2&3 of the original paper, with the exception of being sample-specific over all **pancreas** replicates instead of being organ-specific at each row and column.

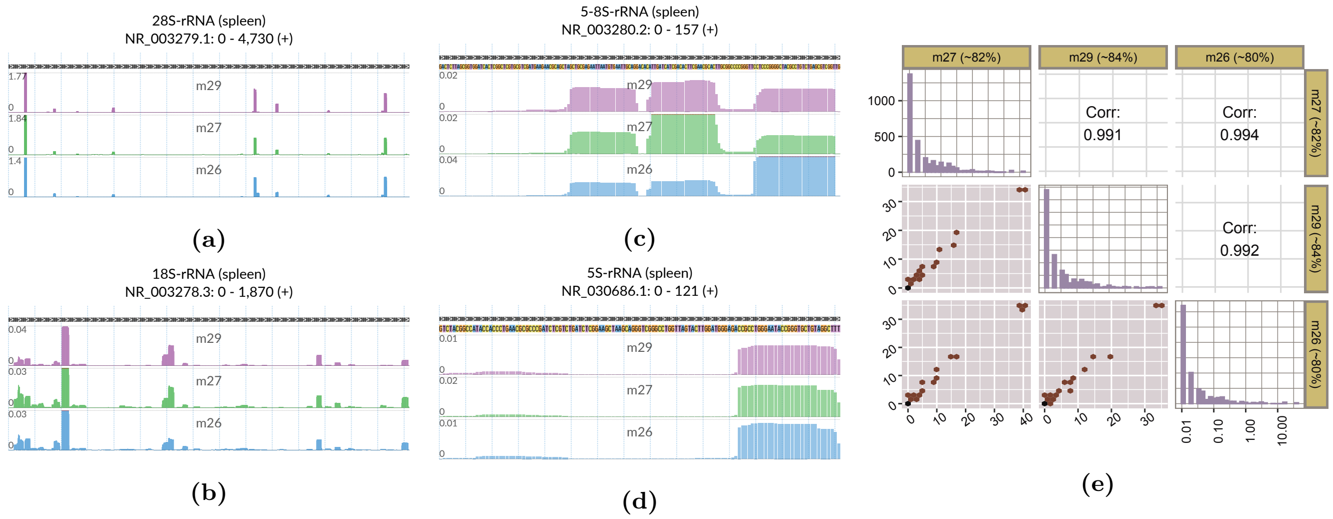

Supplementary Figure 12: Sample-specific rRNA fragment profiles (a-d) of **spleen** replicates and the cross-replicate correlation analysis (e) of oligo depleting potentials computed by *Ribo-ODDR*. These figures are generated in a similar way to Figure 2&3 of the original paper, with the exception of being sample-specific over all **spleen** replicates instead of being organ-specific at each row and column.

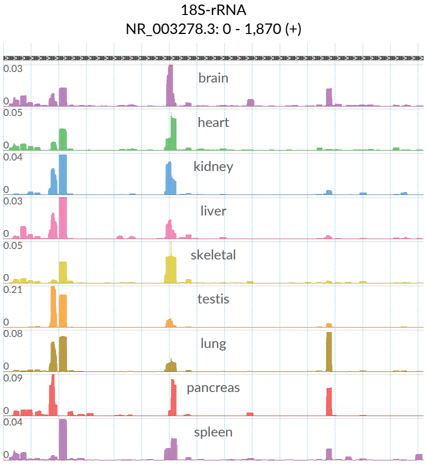

(a)

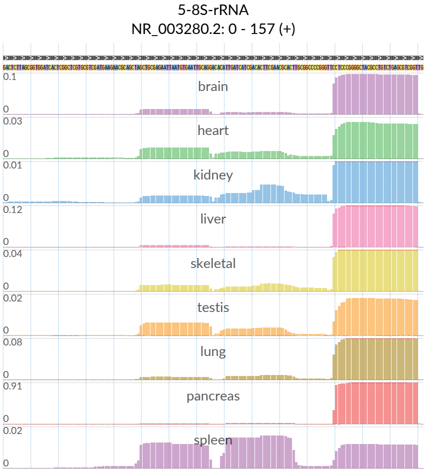

(b)

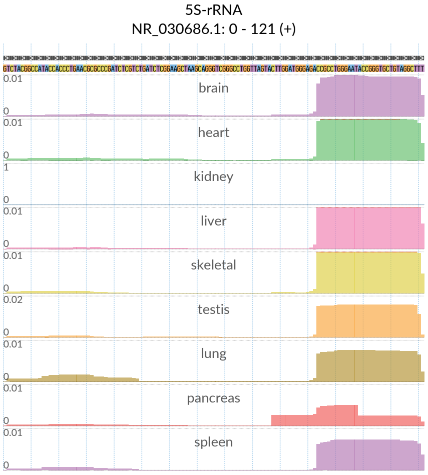

(c)

Supplementary Figure 13: Tissue and RNase specificity of rRNA fragments in mouse Ribo-seq, based on positional abundance profile of 18S (a), 5-8S (b) and 5S (c) rRNA fragments. For every position in the x-axis, y-axis represents the normalized read ratio, number of rRNA reads mapped to that position divided by the total number of reads mapped to all protein coding transcripts.

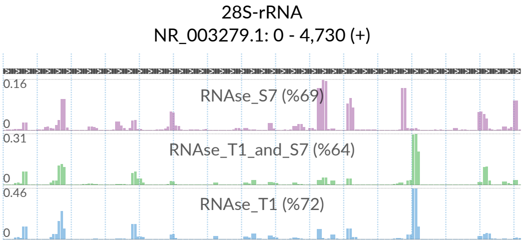

(a)

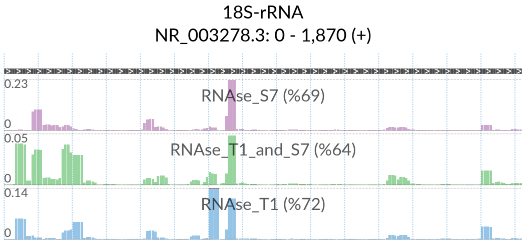

(b)

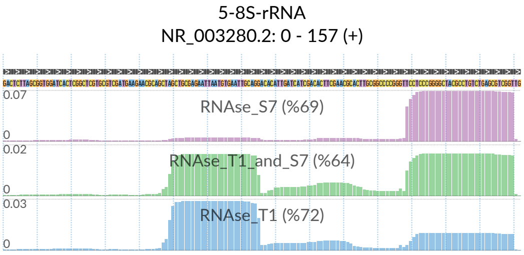

(c)

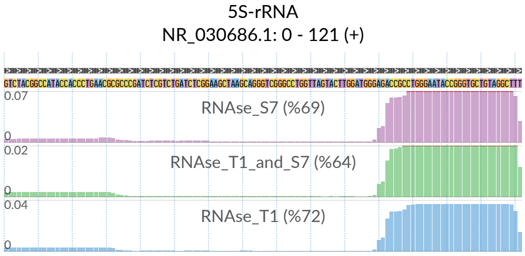

(d)

Supplementary Figure 14: RNase specificity of rRNA fragments in the same tissue, based on *in vivo* Ribo-seq dataset in mouse liver [2]. Figures show the positional abundance profile of 28S(a), 18S (b), 5-8S (c) and 5S (d) rRNA fragments for each analyzed sample. For every position in the x-axis, y-axis represents the normalized read ratio, number of rRNA reads mapped to that position divided by the total number of reads mapped to all protein coding transcripts. Total rRNA percentage of each sample is given in parentheses within the track label.

[2] Maxim V. Gerashchenko, Vadim N. Gladyshev, Ribonuclease selection for ribosome profiling, Nucleic Acids Research, Volume 45, Issue 2, January 2017, Page e6, https://doi.org/10.1093/nar/gkw822

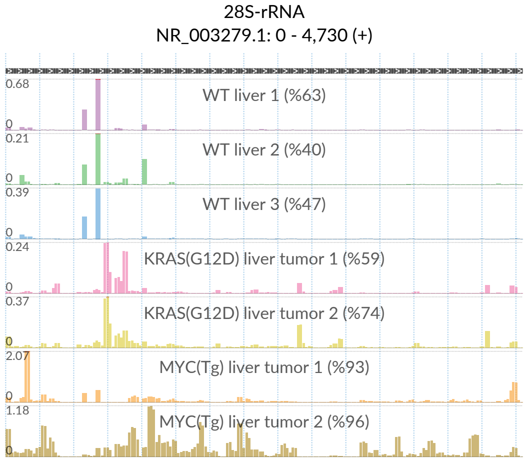

(a)

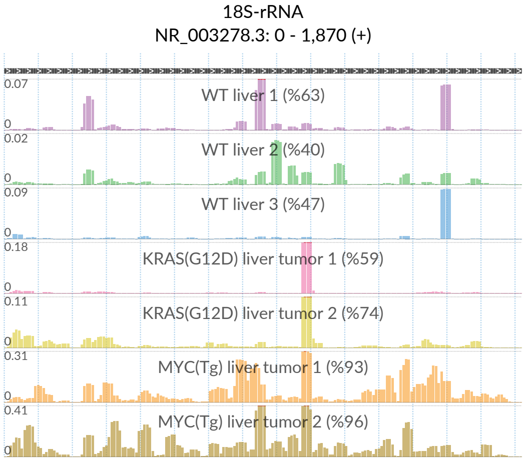

(b)

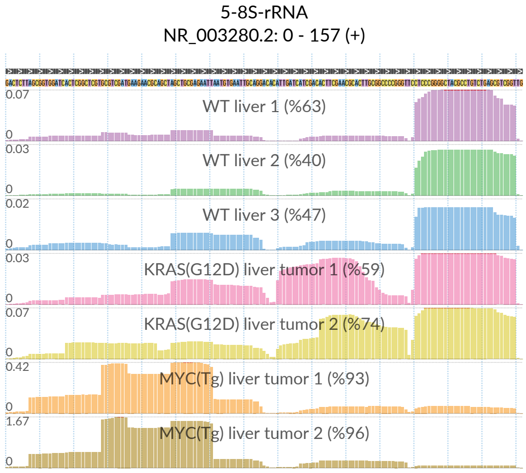

(c)

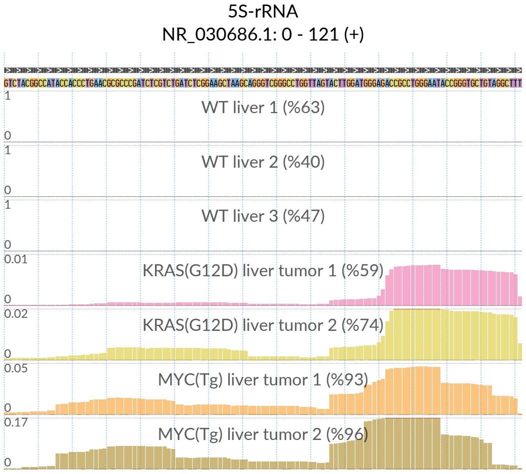

(d)

Supplementary Figure 15: rRNA fragment differences between liver Ribo-seq experiments in mouse with different oncogenic driver mutations. Visualization is based on positional abundance profile of 28S(a), 18S (b), 5-8S (c) and 5S (d) rRNA fragments. For every position in the x-axis, y-axis represents the normalized read ratio, number of rRNA reads mapped to that position divided by the total number of reads mapped to all protein coding transcripts. Total rRNA percentage of each sample is given in parentheses within the track label.

[3] Xu, Y., Poggio, M., Jin, H.Y. et al. Translation control of the immune checkpoint in cancer and its therapeutic targeting. Nat Med 25, 301–311 (2019). https://doi.org/10.1038/s41591-018-0321-2.

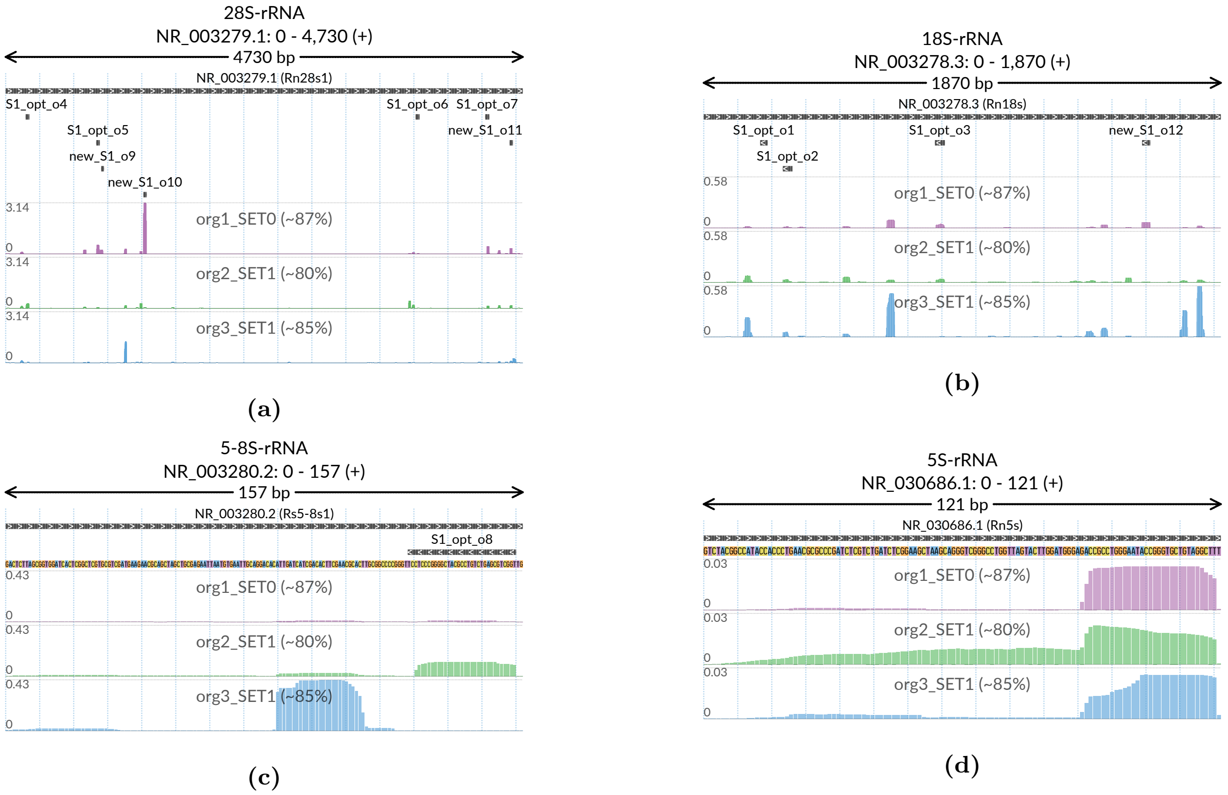

Supplementary Figure 16: Positional abundance profiles of 28S (a), 18S (b), 5-8S (c) and 5S (d) rRNA fragments coming from preliminary *in vitro* Ribo-seq experiments performed with two different sets of rRNA depletion oligos (SET-0 (human oligos) & SET-1). Prior to *in vivo* experiments (in mouse intestine) presented in the paper, we first performed an *in vitro* Ribo-seq (in mouse intestinal organoid) using human rRNA depletion oligos (SET-0, Supplementary Table 1). In this experiment, we observed that $\sim$87% of the reads that mapped to rRNAs and protein-coding transcripts were rRNA fragments and some of these fragments originated from mouse rRNA regions that were actually targeted by human oligos (top row, *org1_SET0*). To improve the overall quality of this experiment, we used the early versions of *Ribo-ODDR cross-species optimization mode* to optimize the human oligos for mouse experiments, and added four new oligos to the pool by manually visualizing rRNA fragments and selecting the hotspots requiring depletion. At the end, the new set of oligos (SET-1) included cross-species optimized version of human oligos together with 4 additional ones (Supplementary Table 2). While this approach did result in increased rRNA depletion, in average only $\sim$18% of sequencing reads mapped to protein coding regions (bottom 2 rows, *org2_SET1* & *org3_SET1*). In the figure, top track indicates the target regions of used oligos within a rRNA, where additional oligos of the SET-1 are labeled as 'new'. In all tracks, x-axis corresponds to position within rRNAs. In sample-specific profile tracks, y-axis is fixed to the same interval and shows the normalized read ratio, number of rRNA reads mapped to the position divided by the total number of reads mapped to all protein coding transcripts. The percentages given within sample labels indicates the sample-specific percentage of rRNA fragments, within all reads that can be mapped to rRNA and protein-coding transcripts.

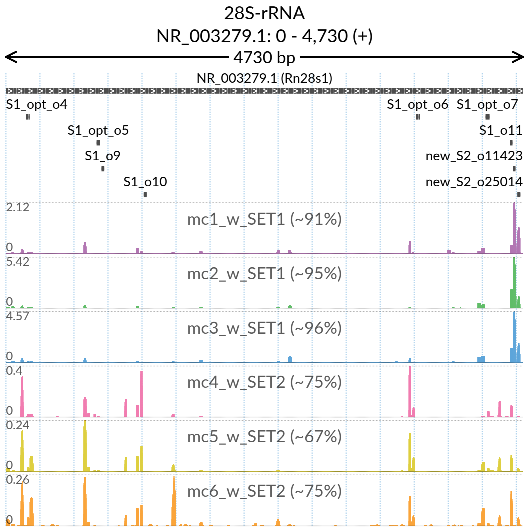

(a)

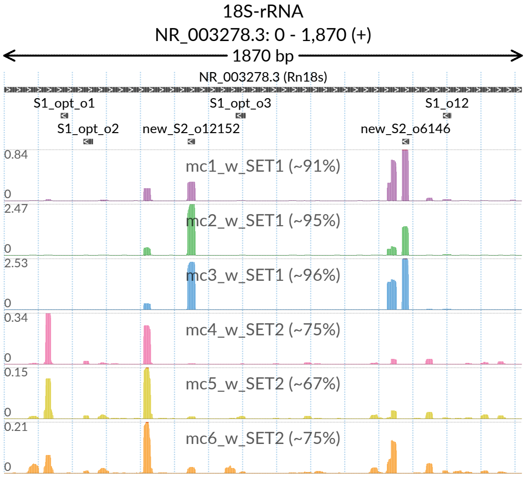

(b)

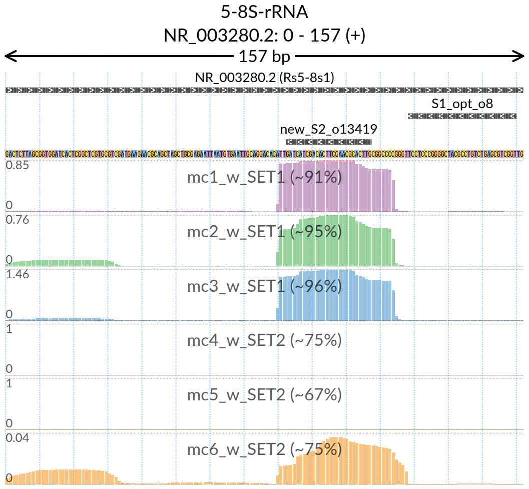

(c)

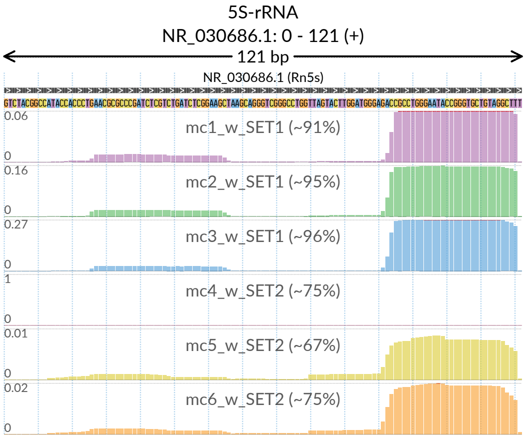

(d)

Supplementary Figure 17: Positional abundance profiles of 28S (a), 18S (b), 5-8S (c) and 5S (d) rRNA fragments coming from *in vivo* (mouse intestine) Ribo-seq experiments performed with two different sets of rRNA depletion oligos (SET-1 & SET-2). SET-2 includes all oligos of the SET-1 together with 5 additional ones, designed with *Ribo-ODDR* based on pilot data generated with experiments using SET-1. In each figure, top track indicates the target regions of used oligos within that rRNA, where additional oligos of the SET-2 are labeled as ‘new’. In all tracks, x-axis corresponds to position within rRNAs. In sample-specific profile tracks, y-axis shows the normalized read ratio, number of rRNA reads mapped to the position divided by the total number of reads mapped to all protein coding transcripts. Y-axis of each row has a different interval between 0 and the max value given in every row. The percentages given within sample labels indicates the sample-specific percentage of rRNA fragments, within all reads that can be mapped to rRNA and protein-coding transcripts.

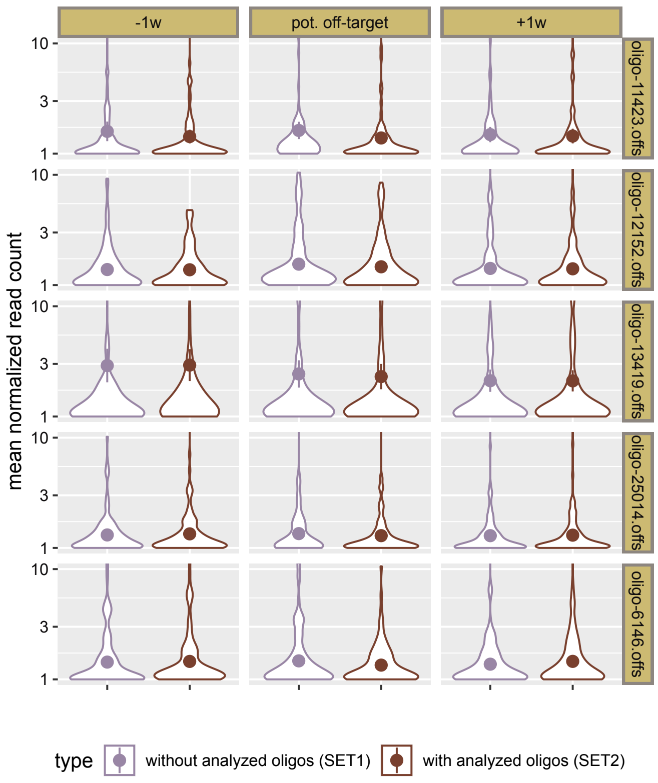

(a)

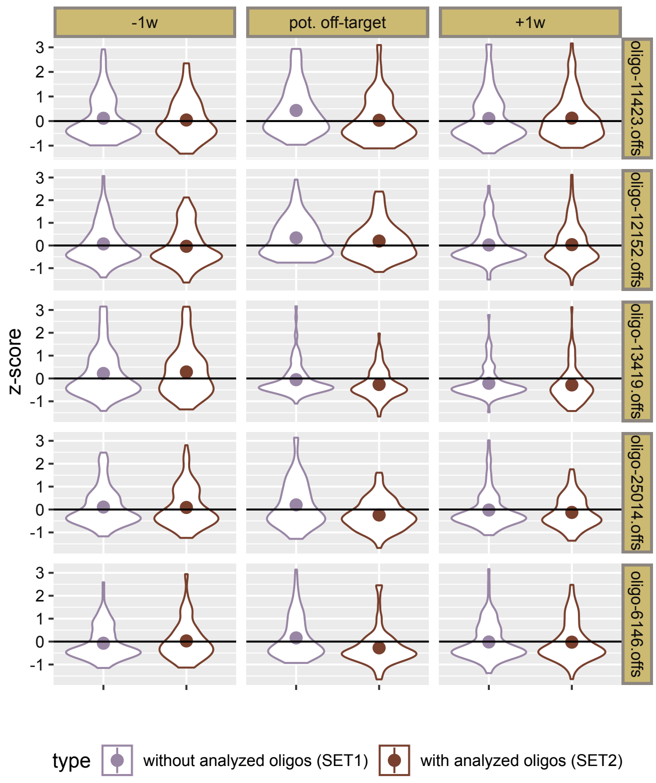

(b)

Supplementary Figure 18: Read count analysis for potential off-targets of new SET2 oligos. Potential off-targets of 5 new oligos are first identified with *Ribo-ODDR* & RIsearch2[2] tools, choosing the 5′UTR+CDS of gencode vM21 protein-coding transcripts as unintended depletion targets. To avoid multiple representation of predicted off-target regions due to alternative isoforms, only predictions within primary transcripts are kept. Later, predicted off-target regions, their upstream and downstream regions (5 x off-target region length) are queried for active ribosome protected fragments (RPFs). If no RPF is found in these regions, that off-target is considered not translated and discarded from analysis. All remaining off-target predictions are ranked based on minimum free energy, representing the likelihood of undesired off-target depletion in the suspected region. From this ranking, we select the top 100 off-target predictions for each oligo to perform the read-count analysis. In the analysis, for each of the 500 selected off-target prediction, we first determine the mean normalized read count mapped to each off-target region and their 5 upstream and 5 downstream neigboring regions (same length as the off-target). This is done separately for *in vivo* Ribo-seq experiments performed with SET-1 and SET-2 oligos. This yields to 11 values per oligo off-target prediction and experiment group, each representing the mean normalized read count of a region (off-target or neigbor) within the experiments using one of the two oligo sets. We store these values not only as it is but also by transforming them into z-scores based on the distribution of these 11 values. In the figure above, on the left (a), we first present how original mean normalized read counts are distributed for SET-1 and SET-2 experiments (different colors), within off-target regions and their immediate neigbors (columns), seperately for each oligo analyzed (rows). The candidate region for unintended depletion is highlighted with red lines and neighboring regions serve as control. On the right (b), instead of original scores, we present, in the same way, the distribution of z-scores, calculated seperately for each oligo off-target prediction and experiment group. In both figures, one can see that reads within the predicted off-target regions (center column) do not deplete due to unintended oligo targeting, having similar distributions between SET-1 and SET-2 experiments, just like as expected for their neigboring regions (left and right columns).

[4] Alkan F, Wenzel A, Palasca O, Kerpedjiev P, Rudebeck AF, Stadler PF, Hofacker IL, Gorodkin J. RIsearch2: suffix array-based large-scale prediction of RNA-RNA interactions and siRNA off-targets. Nucleic Acids Res. 2017 05; 45(8):e60.

type	sequence (5′ –> 3′)
19 nt size selection marker	AGUGUACUCCGAAGAGGAC
32 nt size selection marker	GGCAUUAACGCGAACUCGGCCUACAAUAGUGA
5′ adapter	GUUCAGAGUUCUACAGUCCGACGAUC
3′ adapter	5rApp/UGGAAUUCUCGGGUGCCAAGG/3ddC
RTP	GCCTTGGCACCCGAGAATTCCA
RP1 forward	AATGATACGGCGACCACCGAGATCTACACGTTCAGAGTTCTACAGTCCGA
RPI1 reverse	CAAGCAGAAGACGGCATACGAGATCGTGATGTGACTGGAGTTCCTTGGCACCCGAGAATTCCA
RPI2 reverse	CAAGCAGAAGACGGCATACGAGATACATCGGTGACTGGAGTTCCTTGGCACCCGAGAATTCCA
RPI3 reverse	CAAGCAGAAGACGGCATACGAGATGCCTAAGTGACTGGAGTTCCTTGGCACCCGAGAATTCCA
RPI4 reverse	CAAGCAGAAGACGGCATACGAGATTGGTCAGTGACTGGAGTTCCTTGGCACCCGAGAATTCCA
RPI5 reverse	CAAGCAGAAGACGGCATACGAGATCACTGTGTGACTGGAGTTCCTTGGCACCCGAGAATTCCA
RPI6 reverse	CAAGCAGAAGACGGCATACGAGATATTGGCGTGACTGGAGTTCCTTGGCACCCGAGAATTCCA
RPI7 reverse	CAAGCAGAAGACGGCATACGAGATGATCTGGTGACTGGAGTTCCTTGGCACCCGAGAATTCCA
RPI8 reverse	CAAGCAGAAGACGGCATACGAGATTCAAGTGTGACTGGAGTTCCTTGGCACCCGAGAATTCCA

Supplementary Table 1: Size selection marker, adapter and primer sequences (5′ –> 3′) for experimental methods.

Oligo ID	sequence (5′ –> 3′)	target rRNA
human-o1	UGAUCUGAUAAAUGCCGCAUCCCCCC	18S
human-o2	CGUGCGAUCGGCCCGAGGUUAUCUAGAGUCACCAA	18S
human-o3	AUUCCAUUAUUCCUAGCUGCGGUAUCCAGGCGGCUC	18S
human-o4	GGGCCUCGAUCAGAAGGACUUGGGCCCCCCACGA	28S
human-o5	UCGCUCCCUCGGCCCCGGGAUUCGGCGAGUGC	28S
human-o6	CCGGGCGCUUGGCGCCAGAAGCGAGAGCCCCUCGGG	28S
human-o7	GACCGGCUAUCCGAGGCCAACCGAGGCUCCGCGGCG	28S
human-o8	AGCGACGCUCAGACAGGCGUAGCCCCGGGAGGA	5.8S

Supplementary Table 2: Oligo sequences (5′ –> 3′) for human oligos (SET-0).

Oligo ID	sequence (5′ –> 3′)	target rRNA	target pos.
S1_opt-o1	UGAUCUGAUAAAUGCACGCAUCCCCCC	18S	205-231
S1_opt-o2	CGUGCGAUCGGCCCGAGGUUAUCUAGAGUCACCAA	18S	287-321
S1_opt-o3	AUUCCAUUAUUCCUAGCUGCGGUAUCCAGGCGGCUC	18S	837-872
S1_opt-o4	GGGCCUCGAUCAGAAGGACUUGGGCCCCCCACGA	28S	184-217
S1_opt-o5	UGGCUUCCUCGGCCCCGGGAUUCGGCGAAAGC	28S	830-861
S1_opt-o6	ACGGACGCUUGGCGCCAGAAGCGAGAGCCCCUCGGG	28S	3751-3786
S1_opt-o7	ACCCGGCUAUCCGGGGCCAACCGAGGCUCCUUCGGCG	28S	4388-4424
S1_opt-o8	ACCGACGCUCAGACAGGCGUAGCCCCGGGAGGA	5.8S	123-155
**new_S1_o9**	GGCGGACGGGGGGAGAGGGAGAGCGC	28S	875-900
**new_S1_o10**	GGCGAGACGGGCCGGUGGUGCGCCCUCGGC	28S	1261-1290
**new_S1_o11**	CCAGAAGCAGGUCGUCUACGAAUGGUUUAG	28S	4611-4640
**new_S1_o12**	AUCCCCGAUCCCCAUCACGAAUGGGGUUCA	18S	1586-1615

Supplementary Table 3: Oligo sequences (5′ –> 3′) for oligos in SET-1.

Oligo ID	sequence ($5' \text{-}> 3'$)	target rRNA	target pos.
S1_opt-o1	UGAUCUGAUAAAUGCACGCAUCCCCCC	18S	205-231
S1_opt-o2	CGUGCGAUCGGCCCGAGGUUAUCUAGAGUCACCAA	18S	287-321
S1_opt-o3	AUUCCAUUAUUCCUAGCUGCGGUAUCCAGGCGGCUC	18S	837-872
S1_opt-o4	GGGCCUCGAUCAGAAGGACUUGGGCCCCCCACGA	28S	184-217
S1_opt-o5	UGGCUUCCUCGGCCCCGGGAUUCGGCGAAAGC	28S	830-861
S1_opt-o6	ACGGACGCUUGGCGCCAGAAGCGAGAGCCCCUCGGG	28S	3751-3786
S1_opt-o7	ACCCGGCUAUCCGGGGCCAACCGAGGCUCCUUCGGCG	28S	4388-4424
S1_opt-o8	ACCGACGCUCAGACAGGCGUAGCCCCGGGAGGA	5.8S	123-155
S1_o9	GGCGGACGGGGGGAGAGGGAGAGCGC	28S	875-900
S1_o10	GGCGAGACGGGCCGGUGGUGCGCCCUCGGC	28S	1261-1290
S1_o11	CCAGAAGCAGGUCGUCUACGAAUGGUUUAG	28S	4611-4640
S1_o12	AUCCCCGAUCCCCAUCACGAAUGGGGUUCA	18S	1586-1615
**new_S2_o11423**	GCUCUGCUACGUACGAAACCCCGACC	28S	4640-4665
**new_S2_o25014**	GUGUCGAGGGCUGACUUUCAAUAGAUCG	28S	4679-4706
**new_S2_o12152**	AGAUCCAACUACGAGCUUUUUAACUG	18S	664-689
**new_S2_o6146**	AACGCCACUUGUCCCUCUAAGAAGU	18S	1439-1463
**new_S2_o13419**	CAAGUGCGUUCGAAGUGUCGAUGAUC	5-8S	86-111

Supplementary Table 4: Oligo sequences ($5' \text{-}> 3'$) for oligos in SET-2.

Oligo ID	sequence ($5' \text{-}> 3'$)	target rRNA	target pos.
S1_opt-o1	UGAUCUGAUAAAUGCACGCAUCCCCCC	18S	205-231
S1_opt-o2	CGUGCGAUCGGCCCGAGGUUAUCUAGAGUCACCAA	18S	287-321
S1_opt-o3	AUUCCAUUAUUCCUAGCUGCGGUAUCCAGGCGGCUC	18S	837-872
S1_opt-o4	GGGCCUCGAUCAGAAGGACUUGGGCCCCCCACGA	28S	184-217
S1_opt-o5	UGGCUUCCUCGGCCCCGGGAUUCGGCGAAAGC	28S	830-861
S1_opt-o6	ACGGACGCUUGGCGCCAGAAGCGAGAGCCCCUCGGG	28S	3751-3786
S1_opt-o7	ACCCGGCUAUCCGGGGCCAACCGAGGCUCCUUCGGCG	28S	4388-4424
S1_opt-o8	ACCGACGCUCAGACAGGCGUAGCCCCGGGAGGA	5.8S	123-155
S1_o9	GGCGGACGGGGGGAGAGGGAGAGCGC	28S	875-900
S1_o10	GGCGAGACGGGCCGGUGGUGCGCCCUCGGC	28S	1261-1290
S1_o11	CCAGAAGCAGGUCGUCUACGAAUGGUUUAG	28S	4611-4640
S1_o12	AUCCCCGAUCCCCAUCACGAAUGGGGUUCA	18S	1586-1615
S2_o11423	GCUCUGCUACGUACGAAACCCCGACC	28S	4640-4665
S2_o25014	GUGUCGAGGGCUGACUUUCAAUAGAUCG	28S	4679-4706
S2_o12152	AGAUCCAACUACGAGCUUUUUAACUG	18S	664-689
S2_o6146	AACGCCACUUGUCCCUCUAAGAAGU	18S	1439-1463
S2_o13419	CAAGUGCGUUCGAAGUGUCGAUGAUC	5-8S	86-111
**new_S3_o6924**	GUCUUCCGUACGCCACAUUUCCCACG	28S	141-166
**new_S3_o35101**	GACUGGAGAGGCCUCGGGAUCCCACCUCGG	28S	1230-1259
**new_S3_o10474**	ACGAUGAGAGUAGUGGUAUUUCACCG	28S	3691-3716
**new_S3_o25177**	UCUAGAAUUACCACAGUUAUCCAAGUAG	18S	139-166
**new_S3_o5218**	CUGUAUUGUUAUUUUUCGUCACUAC	18S	511-535

Supplementary Table 5: Oligo sequences ($5' \text{-}> 3'$) for oligos in SET-3.

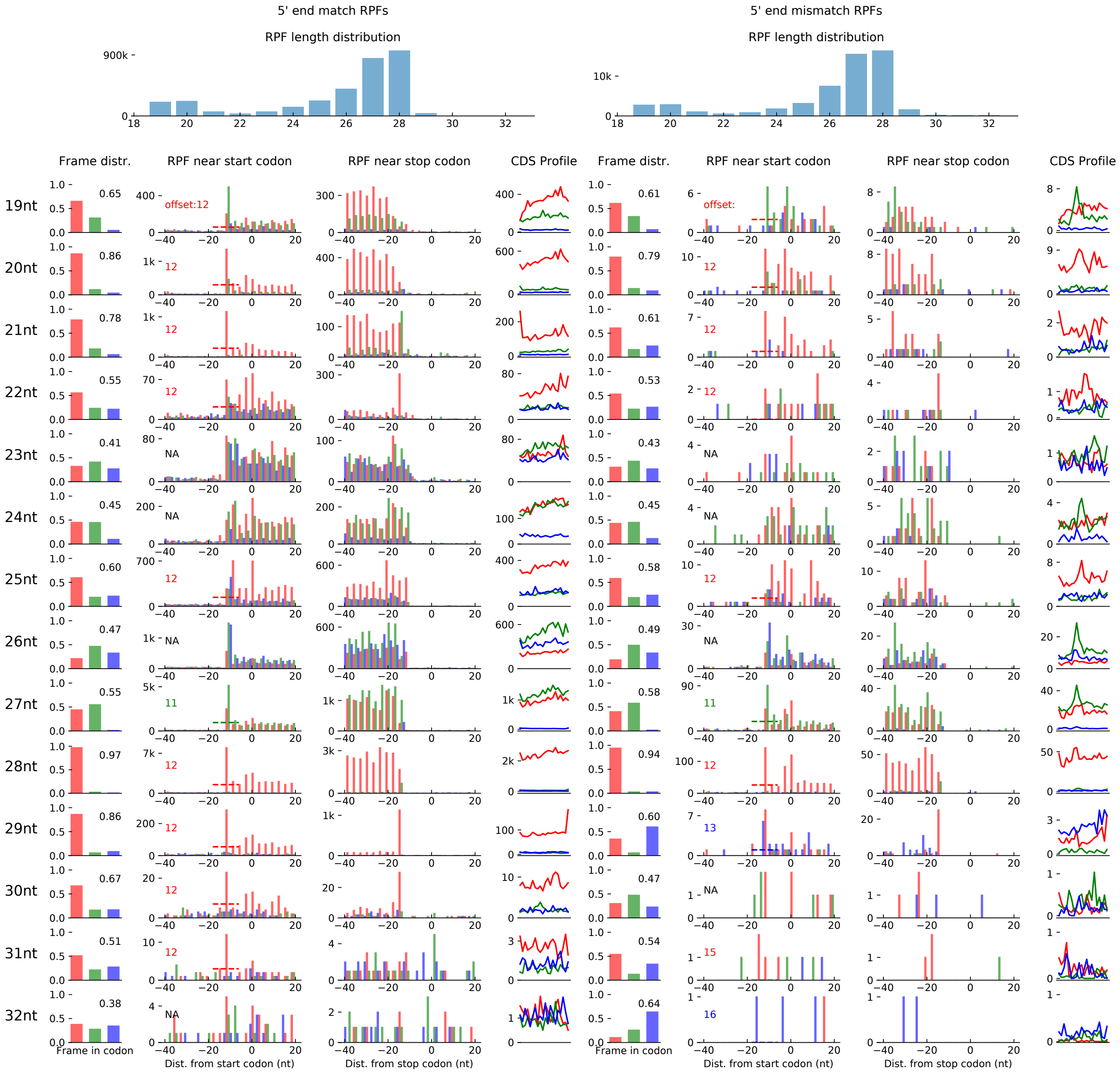

Supplementary Figure 19: Quality Control plots for the Ribo-seq experiment **mc1_w_SET1**, generated by Ribo-TISH tool [3]. By default, QC plots are created seperately for 5′ end matching (left panel) and mismatching (right panel) protein-coding gene mapped reads. In each panel, top figure shows the histogram of read lengths. Below, for each read length between 19 and 32 nts, one can see, from left-to-right, the frequency of in&out of frame reads, start codon periodicity, stop codon periodicity and CDS mapping profile of reads with that specific length.

[5]Zhang, P., He, D., Xu, Y., Hou, J., Pan, B. F., Wang, Y., Liu, T., Davis, C. M., Ehli, E. A., Tan, L., Zhou, F., Hu, J., Yu, Y., Chen, X., Nguyen, T. M., Rosen, J. M., Hawke, D. H., Ji, Z. & Chen, Y. 2017. Genome-wide identification and differential analysis of translational initiation. Nat Commun, 8, 1749.

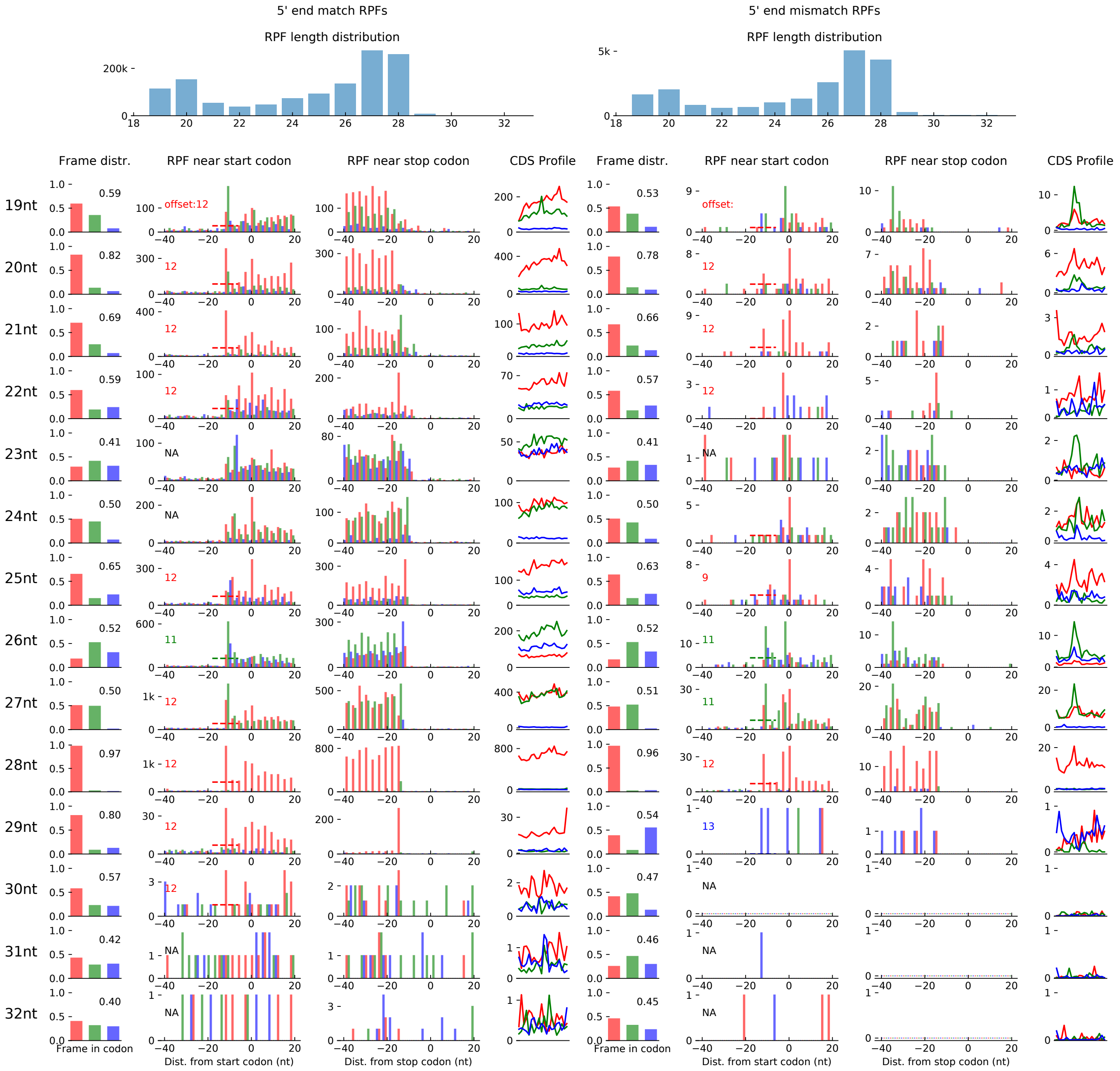

Supplementary Figure 20: Quality Control plots for the Ribo-seq experiment **mc2_w_SET1**, generated by Ribo-TISH tool [3]. By default, QC plots are created seperately for 5′ end matching (left panel) and mismatching (right panel) protein-coding gene mapped reads. In each panel, top figure shows the histogram of read lengths. Below, for each read length between 19 and 32 nts, one can see, from left-to-right, the frequency of in&out of frame reads, start codon periodicity, stop codon periodicity and CDS mapping profile of reads with that specific length.

[5] Zhang, P., He, D., Xu, Y., Hou, J., Pan, B. F., Wang, Y., Liu, T., Davis, C. M., Ehli, E. A., Tan, L., Zhou, F., Hu, J., Yu, Y., Chen, X., Nguyen, T. M., Rosen, J. M., Hawke, D. H., Ji, Z. & Chen, Y. 2017. Genome-wide identification and differential analysis of translational initiation. Nat Commun, 8, 1749.

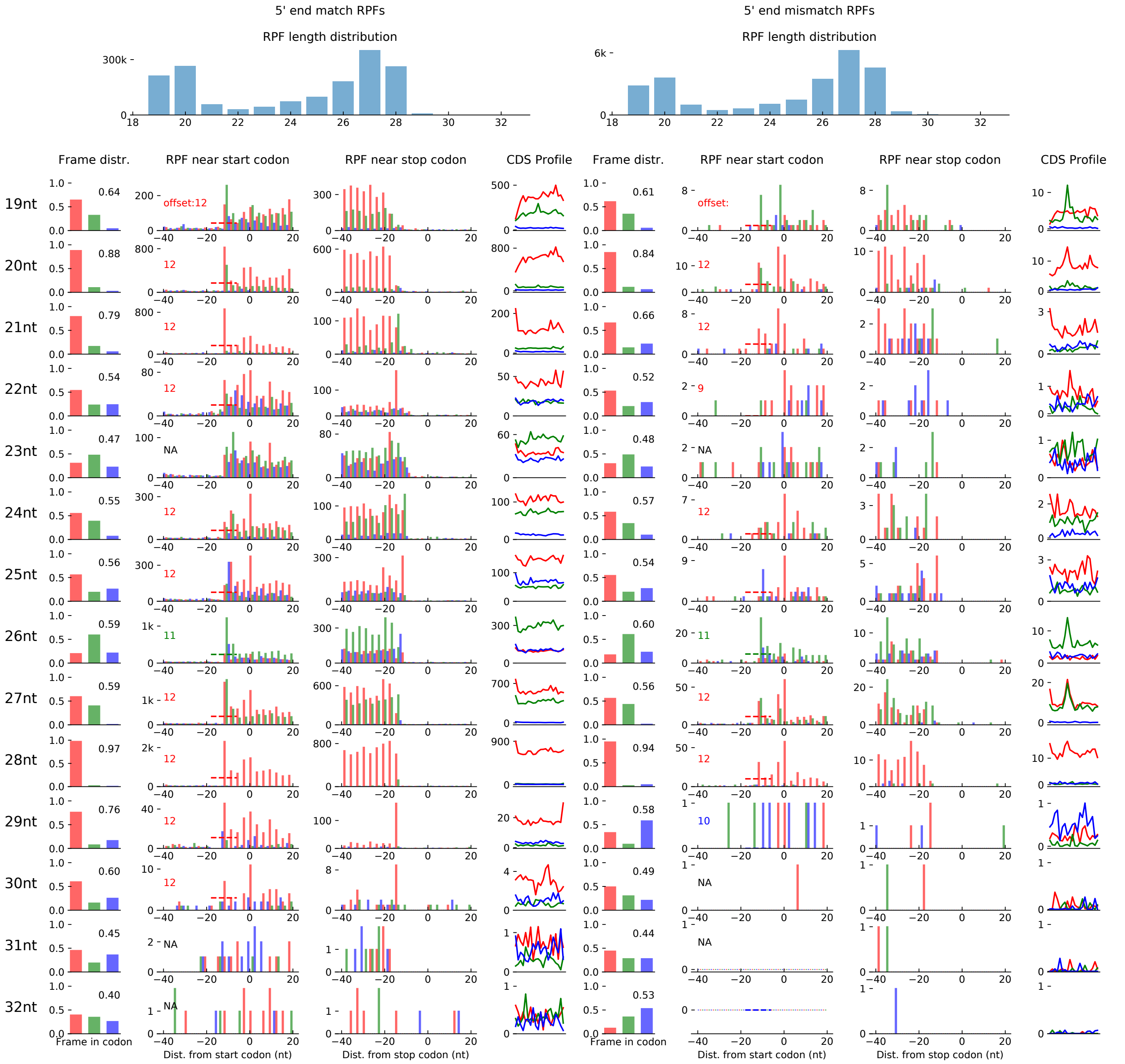

Supplementary Figure 21: Quality Control plots for the Ribo-seq experiment **mc3_w_SET1**, generated by Ribo-TISH tool [3]. By default, QC plots are created seperately for 5′ end matching (left panel) and mismatching (right panel) protein-coding gene mapped reads. In each panel, top figure shows the histogram of read lengths. Below, for each read length between 19 and 32 nts, one can see, from left-to-right, the frequency of in&out of frame reads, start codon periodicity, stop codon periodicity and CDS mapping profile of reads with that specific length.

[5] Zhang, P., He, D., Xu, Y., Hou, J., Pan, B. F., Wang, Y., Liu, T., Davis, C. M., Ehli, E. A., Tan, L., Zhou, F., Hu, J., Yu, Y., Chen, X., Nguyen, T. M., Rosen, J. M., Hawke, D. H., Ji, Z. & Chen, Y. 2017. Genome-wide identification and differential analysis of translational initiation. Nat Commun, 8, 1749.

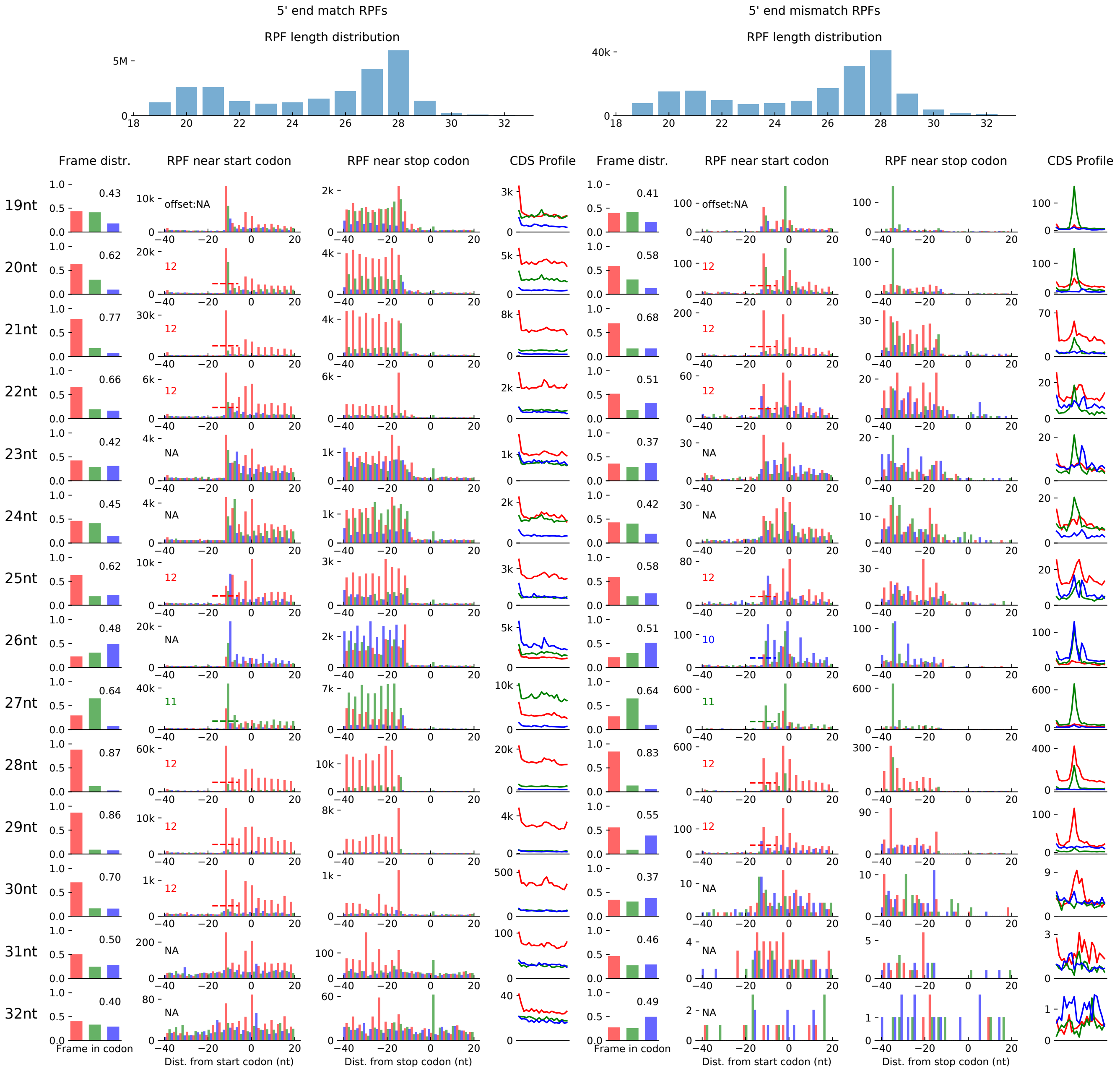

Supplementary Figure 22: Quality Control plots for the Ribo-seq experiment **mc4_w_SET2**, generated by Ribo-TISH tool [3]. By default, QC plots are created seperately for 5′ end matching (left panel) and mismatching (right panel) protein-coding gene mapped reads. In each panel, top figure shows the histogram of read lengths. Below, for each read length between 19 and 32 nts, one can see, from left-to-right, the frequency of in&out of frame reads, start codon periodicity, stop codon periodicity and CDS mapping profile of reads with that specific length.

[5]Zhang, P., He, D., Xu, Y., Hou, J., Pan, B. F., Wang, Y., Liu, T., Davis, C. M., Ehli, E. A., Tan, L., Zhou, F., Hu, J., Yu, Y., Chen, X., Nguyen, T. M., Rosen, J. M., Hawke, D. H., Ji, Z. & Chen, Y. 2017. Genome-wide identification and differential analysis of translational initiation. Nat Commun, 8, 1749.

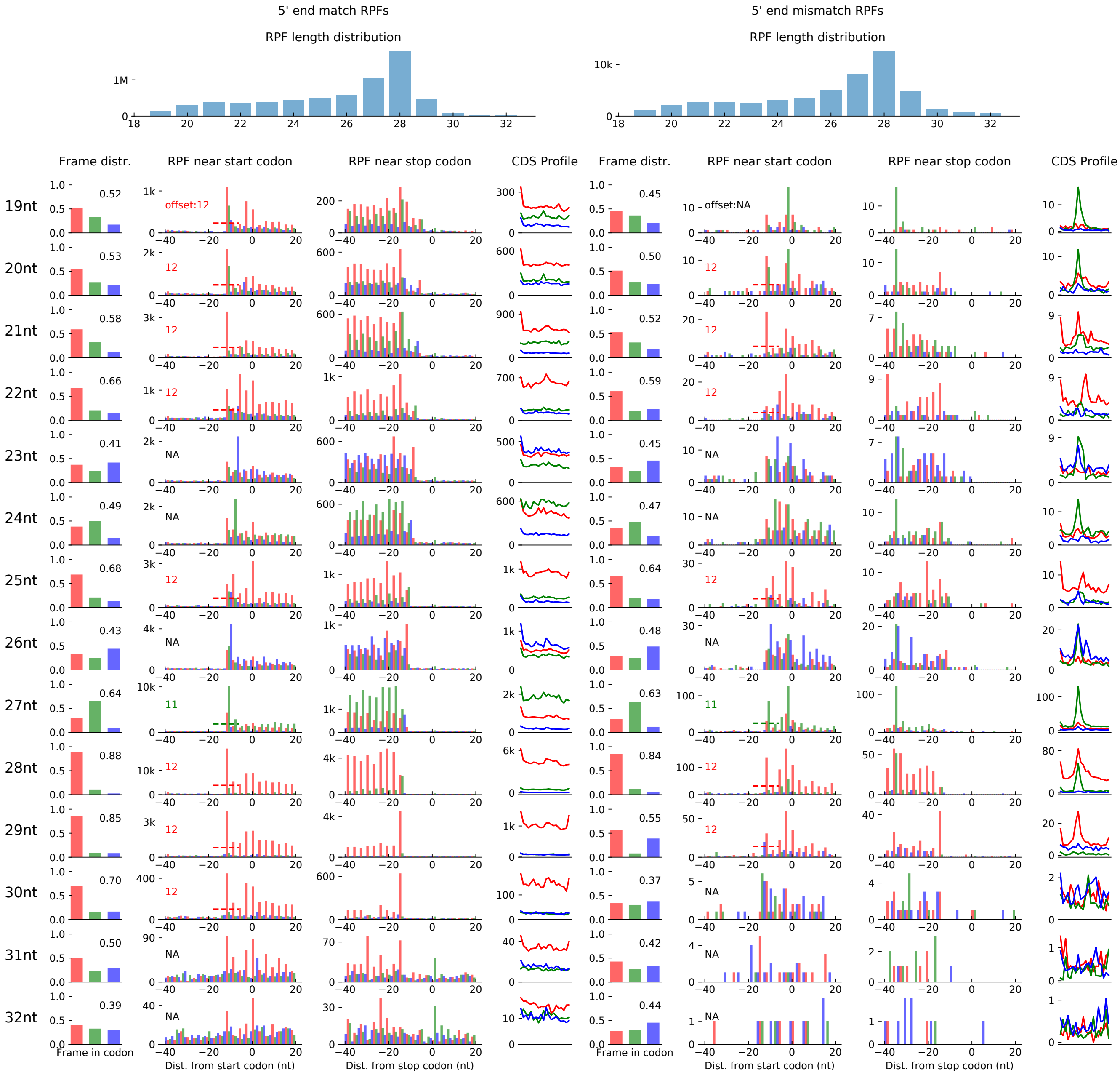

Supplementary Figure 23: Quality Control plots for the Ribo-seq experiment **mc5_w_SET2**, generated by Ribo-TISH tool [3]. By default, QC plots are created seperately for 5′ end matching (left panel) and mismatching (right panel) protein-coding gene mapped reads. In each panel, top figure shows the histogram of read lengths. Below, for each read length between 19 and 32 nts, one can see, from left-to-right, the frequency of in&out of frame reads, start codon periodicity, stop codon periodicity and CDS mapping profile of reads with that specific length.

[5] Zhang, P., He, D., Xu, Y., Hou, J., Pan, B. F., Wang, Y., Liu, T., Davis, C. M., Ehli, E. A., Tan, L., Zhou, F., Hu, J., Yu, Y., Chen, X., Nguyen, T. M., Rosen, J. M., Hawke, D. H., Ji, Z. & Chen, Y. 2017. Genome-wide identification and differential analysis of translational initiation. Nat Commun, 8, 1749.

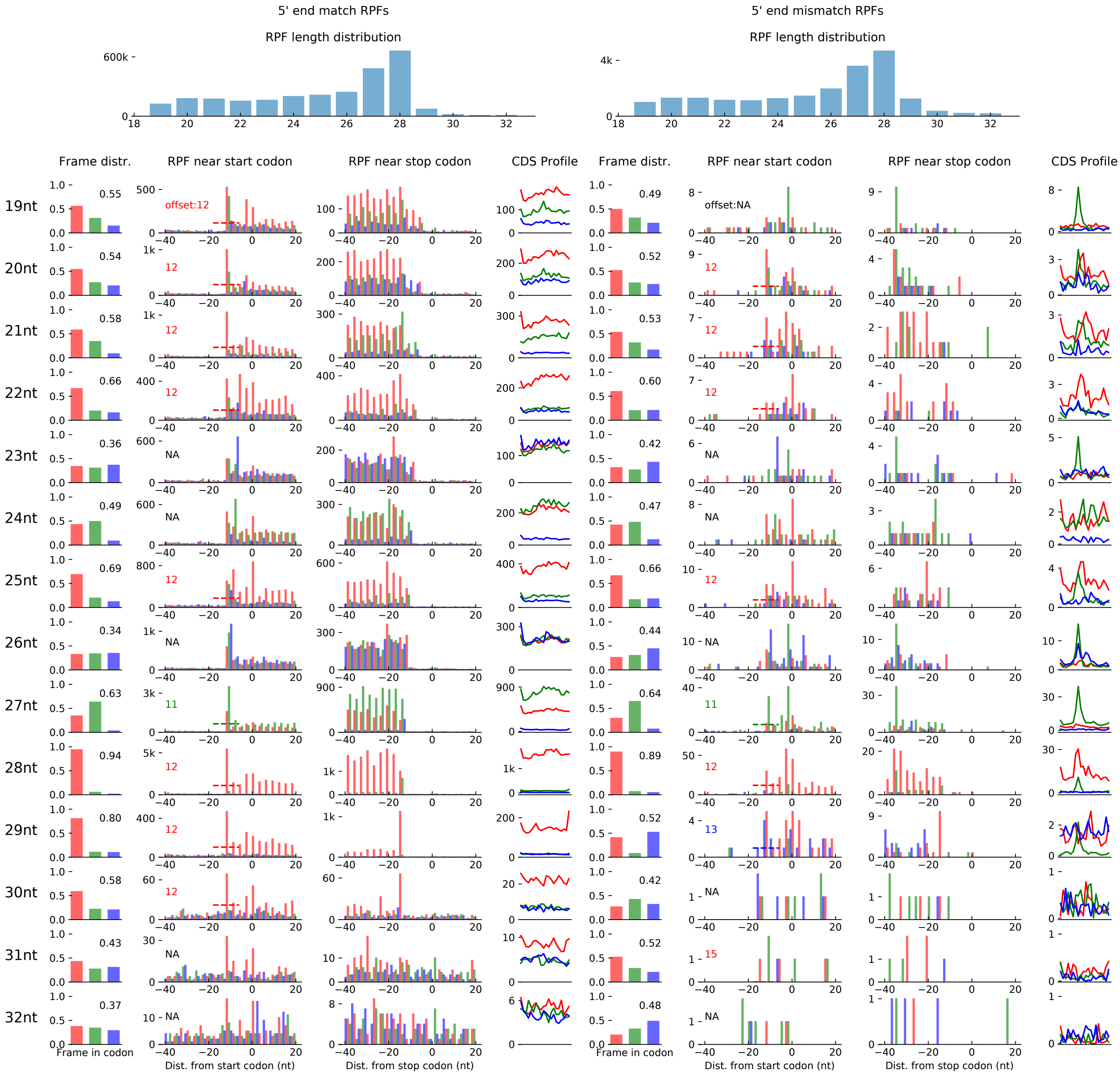

Supplementary Figure 24: Quality Control plots for the Ribo-seq experiment **mc6_w_SET2**, generated by Ribo-TISH tool [3]. By default, QC plots are created seperately for 5′ end matching (left panel) and mismatching (right panel) protein-coding gene mapped reads. In each panel, top figure shows the histogram of read lengths. Below, for each read length between 19 and 32 nts, one can see, from left-to-right, the frequency of in&out of frame reads, start codon periodicity, stop codon periodicity and CDS mapping profile of reads with that specific length.

[5]Zhang, P., He, D., Xu, Y., Hou, J., Pan, B. F., Wang, Y., Liu, T., Davis, C. M., Ehli, E. A., Tan, L., Zhou, F., Hu, J., Yu, Y., Chen, X., Nguyen, T. M., Rosen, J. M., Hawke, D. H., Ji, Z. & Chen, Y. 2017. Genome-wide identification and differential analysis of translational initiation. Nat Commun, 8, 1749.

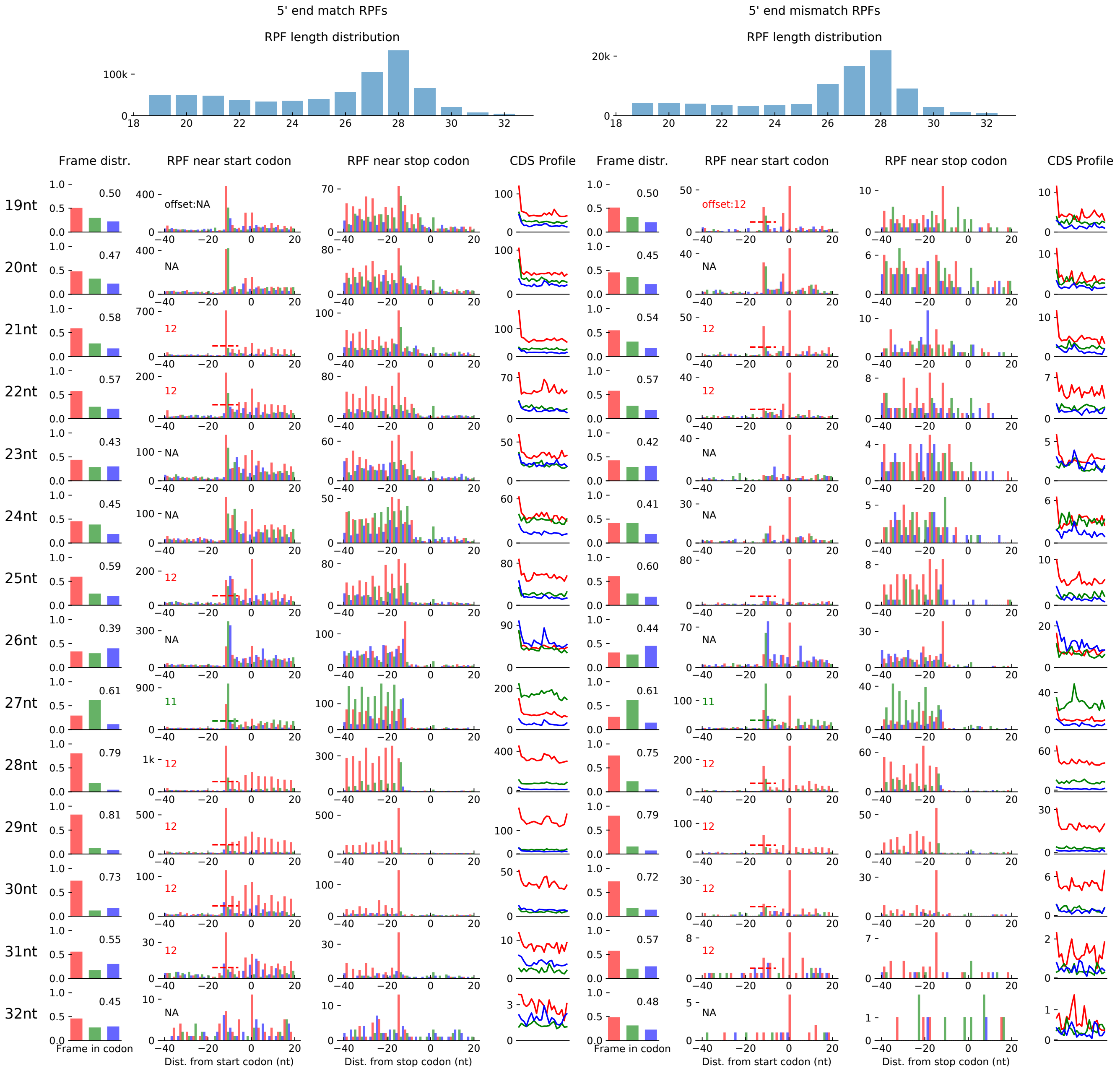

Supplementary Figure 25: Quality Control plots for the Ribo-seq experiment **mc7_w_SET3**, generated by Ribo-TISH tool [3]. By default, QC plots are created seperately for 5′ end matching (left panel) and mismatching (right panel) protein-coding gene mapped reads. In each panel, top figure shows the histogram of read lengths. Below, for each read length between 19 and 32 nts, one can see, from left-to-right, the frequency of in&out of frame reads, start codon periodicity, stop codon periodicity and CDS mapping profile of reads with that specific length.

[5] Zhang, P., He, D., Xu, Y., Hou, J., Pan, B. F., Wang, Y., Liu, T., Davis, C. M., Ehli, E. A., Tan, L., Zhou, F., Hu, J., Yu, Y., Chen, X., Nguyen, T. M., Rosen, J. M., Hawke, D. H., Ji, Z. & Chen, Y. 2017. Genome-wide identification and differential analysis of translational initiation. Nat Commun, 8, 1749.

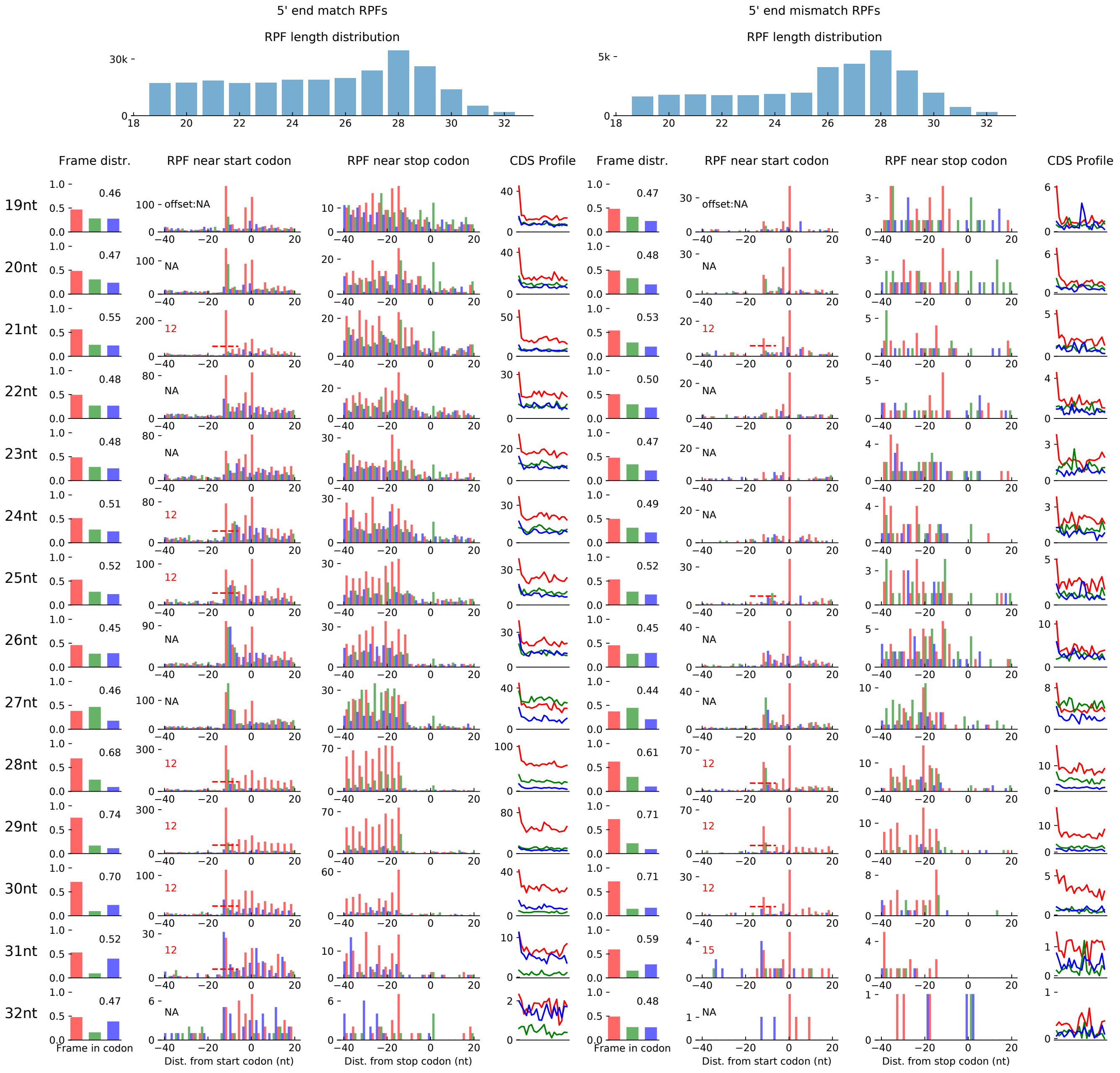

Supplementary Figure 26: Quality Control plots for the Ribo-seq experiment **mc8_w_SET3**, generated by Ribo-TISH tool [3]. By default, QC plots are created seperately for 5′ end matching (left panel) and mismatching (right panel) protein-coding gene mapped reads. In each panel, top figure shows the histogram of read lengths. Below, for each read length between 19 and 32 nts, one can see, from left-to-right, the frequency of in&out of frame reads, start codon periodicity, stop codon periodicity and CDS mapping profile of reads with that specific length.

[5] Zhang, P., He, D., Xu, Y., Hou, J., Pan, B. F., Wang, Y., Liu, T., Davis, C. M., Ehli, E. A., Tan, L., Zhou, F., Hu, J., Yu, Y., Chen, X., Nguyen, T. M., Rosen, J. M., Hawke, D. H., Ji, Z. & Chen, Y. 2017. Genome-wide identification and differential analysis of translational initiation. Nat Commun, 8, 1749.

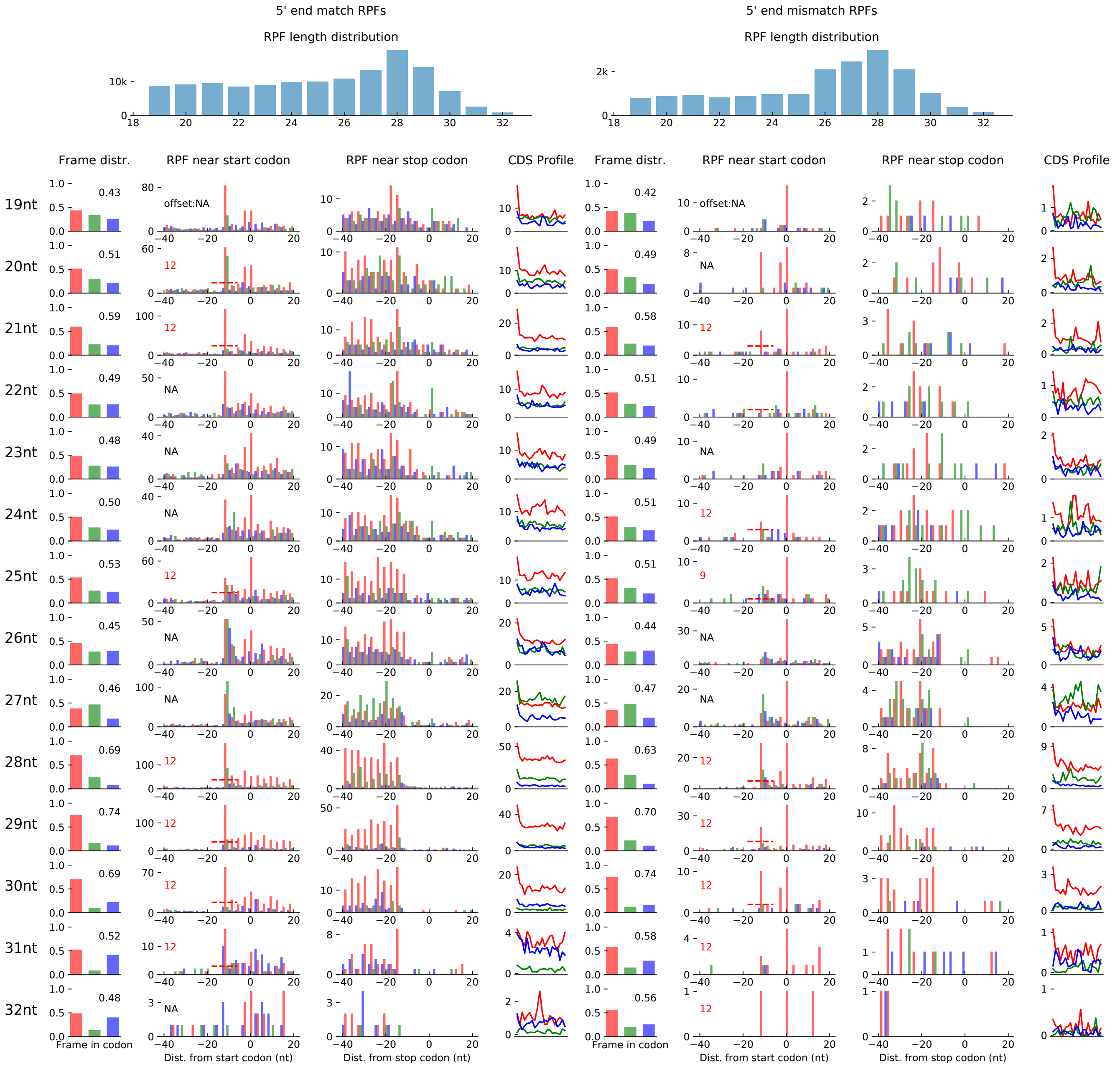

Supplementary Figure 27: Quality Control plots for the Ribo-seq experiment **mc9_RiboZero**, generated by Ribo-TISH tool [3]. By default, QC plots are created seperately for 5′ end matching (left panel) and mismatching (right panel) protein-coding gene mapped reads. In each panel, top figure shows the histogram of read lengths. Below, for each read length between 19 and 32 nts, one can see, from left-to-right, the frequency of in&out of frame reads, start codon periodicity, stop codon periodicity and CDS mapping profile of reads with that specific length.

[5] Zhang, P., He, D., Xu, Y., Hou, J., Pan, B. F., Wang, Y., Liu, T., Davis, C. M., Ehli, E. A., Tan, L., Zhou, F., Hu, J., Yu, Y., Chen, X., Nguyen, T. M., Rosen, J. M., Hawke, D. H., Ji, Z. & Chen, Y. 2017. Genome-wide identification and differential analysis of translational initiation. Nat Commun, 8, 1749.

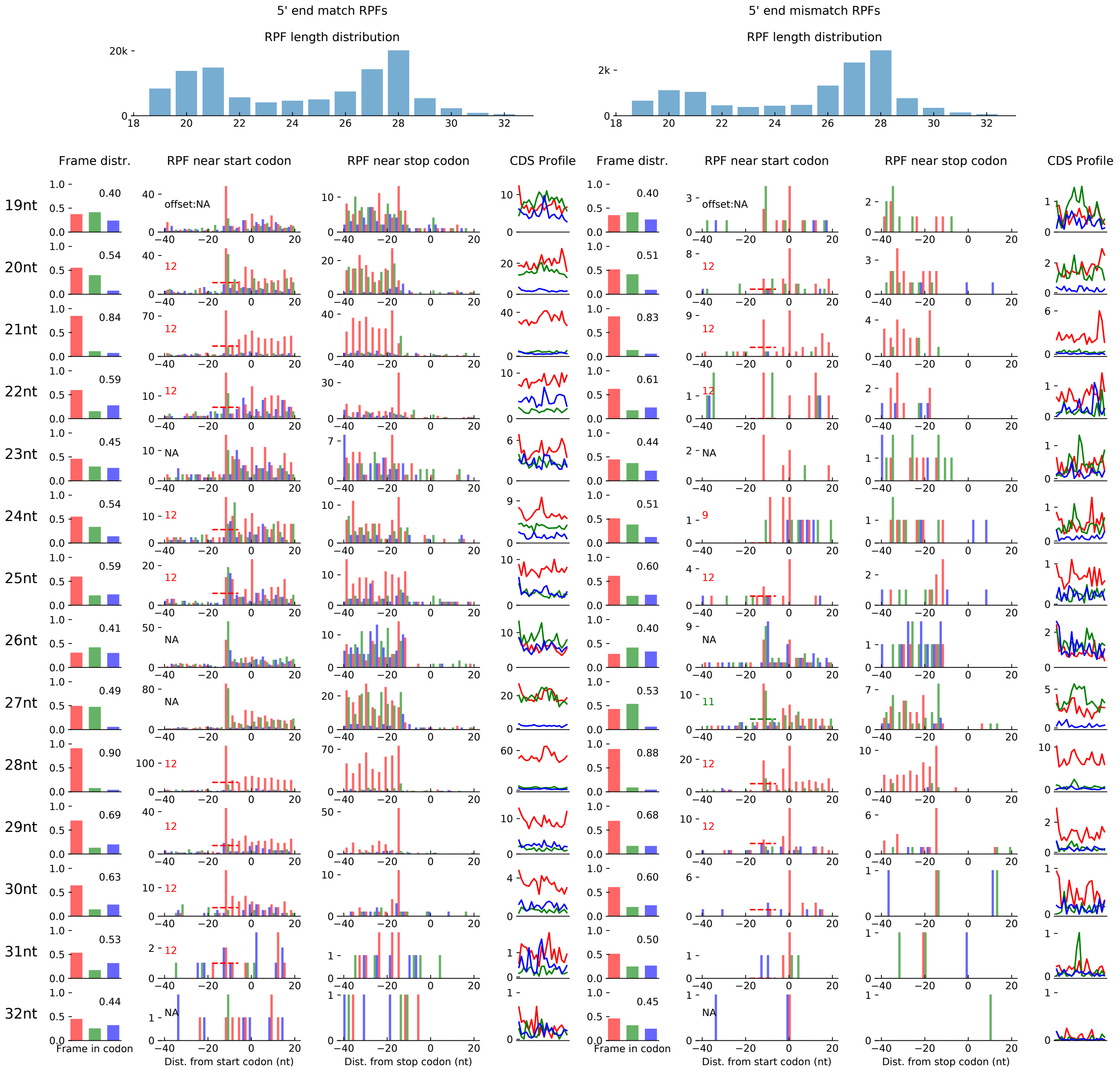

Supplementary Figure 28: Quality Control plots for the Ribo-seq experiment **mc10_RiboZero**, generated by Ribo-TISH tool [3]. By default, QC plots are created seperately for 5′ end matching (left panel) and mismatching (right panel) protein-coding gene mapped reads. In each panel, top figure shows the histogram of read lengths. Below, for each read length between 19 and 32 nts, one can see, from left-to-right, the frequency of in&out of frame reads, start codon periodicity, stop codon periodicity and CDS mapping profile of reads with that specific length.

[5]Zhang, P., He, D., Xu, Y., Hou, J., Pan, B. F., Wang, Y., Liu, T., Davis, C. M., Ehli, E. A., Tan, L., Zhou, F., Hu, J., Yu, Y., Chen, X., Nguyen, T. M., Rosen, J. M., Hawke, D. H., Ji, Z. & Chen, Y. 2017. Genome-wide identification and differential analysis of translational initiation. Nat Commun, 8, 1749.

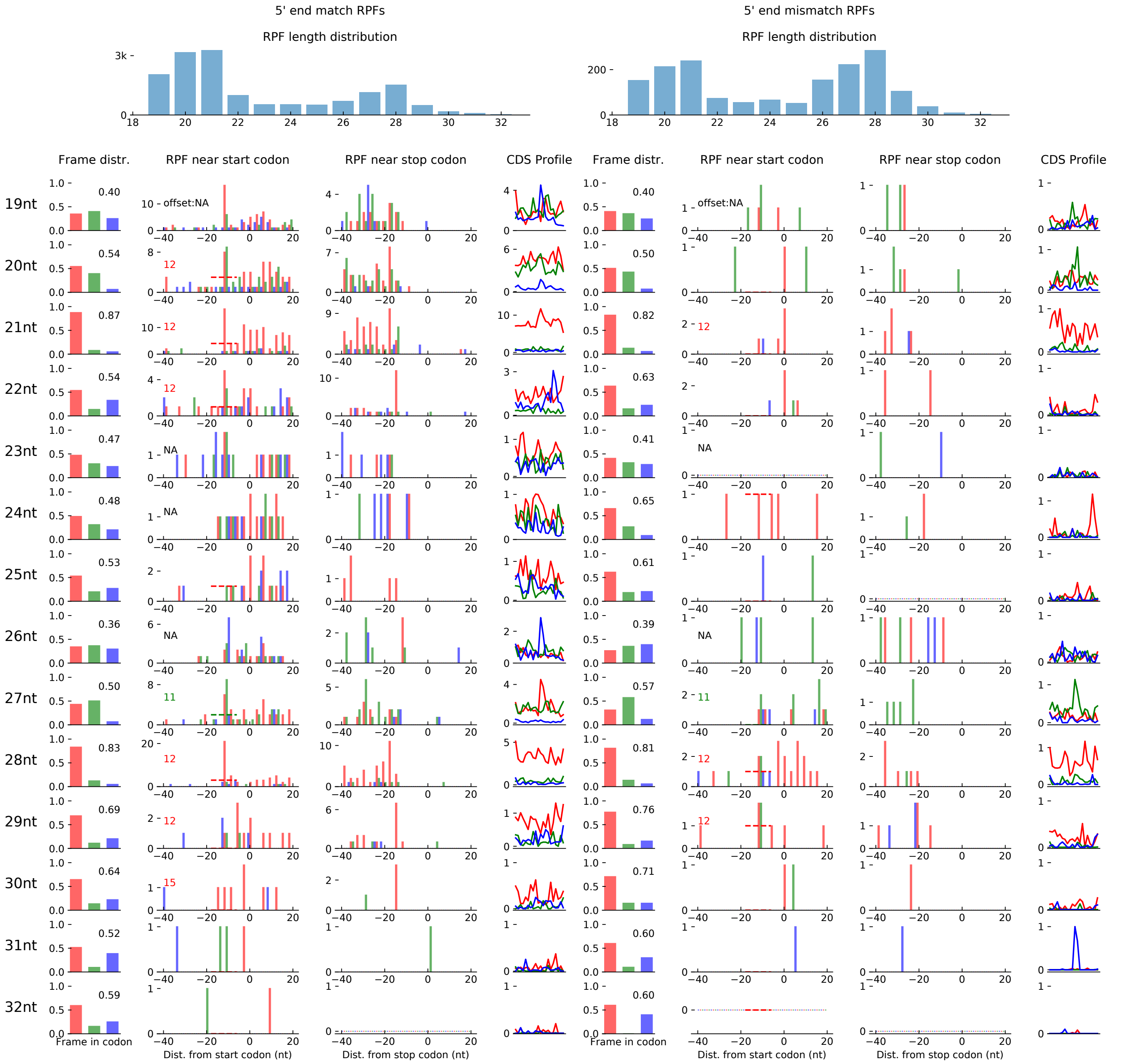

Supplementary Figure 29: Quality Control plots for the Ribo-seq experiment **mc11_RiboCop**, generated by Ribo-TISH tool [3]. By default, QC plots are created seperately for 5′ end matching (left panel) and mismatching (right panel) protein-coding gene mapped reads. In each panel, top figure shows the histogram of read lengths. Below, for each read length between 19 and 32 nts, one can see, from left-to-right, the frequency of in&out of frame reads, start codon periodicity, stop codon periodicity and CDS mapping profile of reads with that specific length.

[5] Zhang, P., He, D., Xu, Y., Hou, J., Pan, B. F., Wang, Y., Liu, T., Davis, C. M., Ehli, E. A., Tan, L., Zhou, F., Hu, J., Yu, Y., Chen, X., Nguyen, T. M., Rosen, J. M., Hawke, D. H., Ji, Z. & Chen, Y. 2017. Genome-wide identification and differential analysis of translational initiation. Nat Commun, 8, 1749.

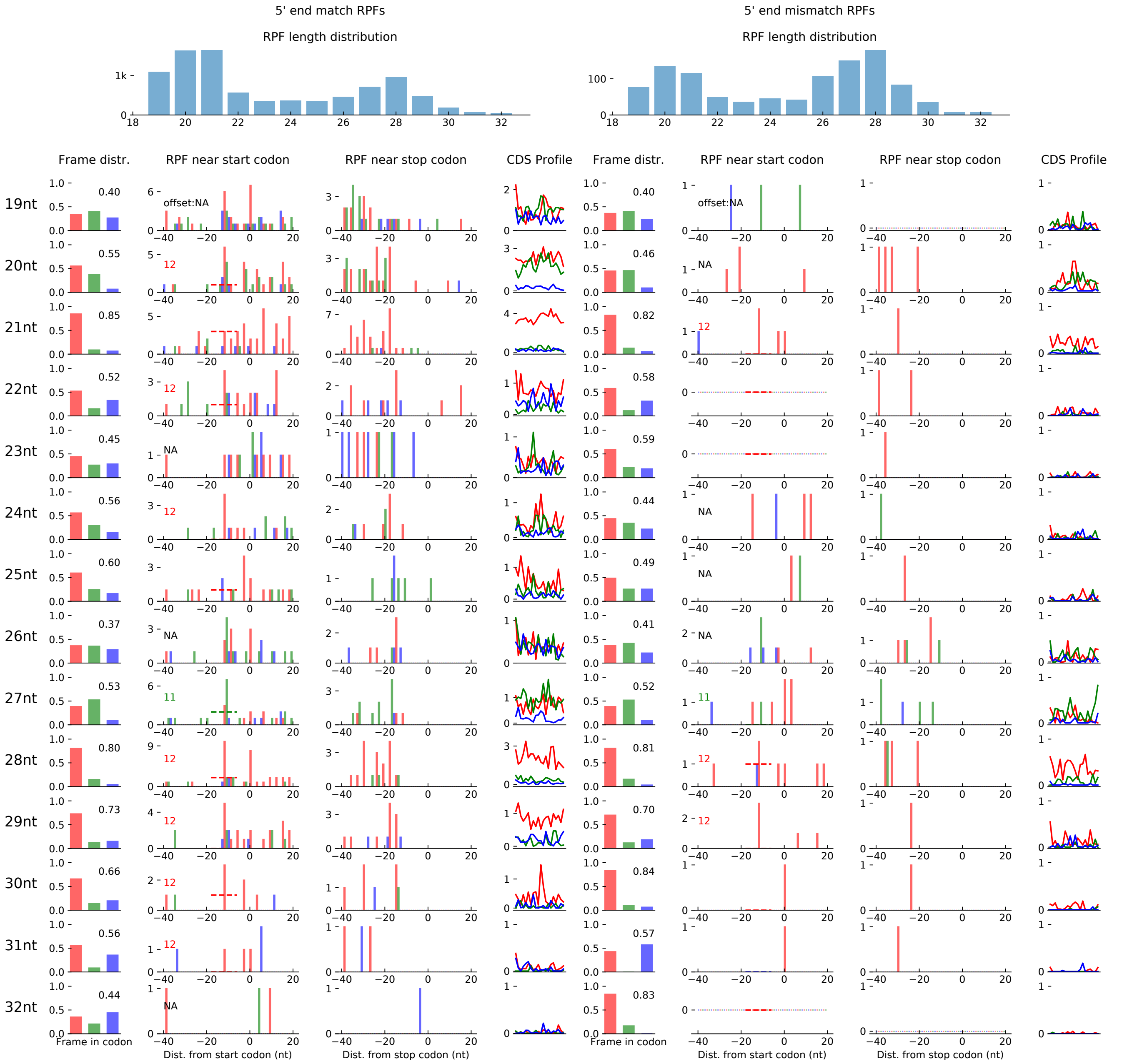

Supplementary Figure 30: Quality Control plots for the Ribo-seq experiment **mc12_RiboCop**, generated by Ribo-TISH tool [3]. By default, QC plots are created seperately for 5′ end matching (left panel) and mismatching (right panel) protein-coding gene mapped reads. In each panel, top figure shows the histogram of read lengths. Below, for each read length between 19 and 32 nts, one can see, from left-to-right, the frequency of in&out of frame reads, start codon periodicity, stop codon periodicity and CDS mapping profile of reads with that specific length.

[5] Zhang, P., He, D., Xu, Y., Hou, J., Pan, B. F., Wang, Y., Liu, T., Davis, C. M., Ehli, E. A., Tan, L., Zhou, F., Hu, J., Yu, Y., Chen, X., Nguyen, T. M., Rosen, J. M., Hawke, D. H., Ji, Z. & Chen, Y. 2017. Genome-wide identification and differential analysis of translational initiation. Nat Commun, 8, 1749.

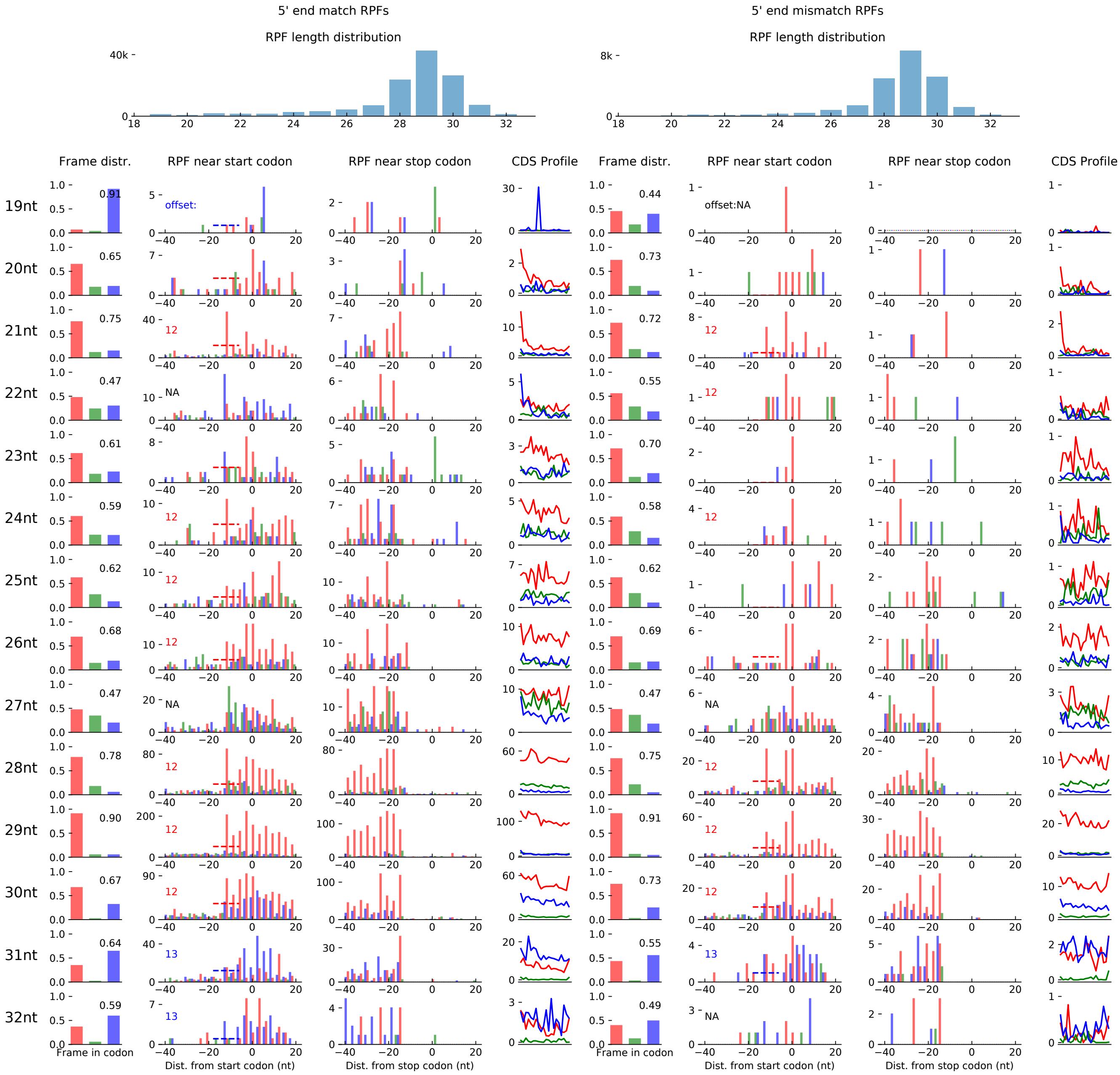

Supplementary Figure 31: Quality Control plots for the Ribo-seq experiment **org1_RiboCop**, generated by Ribo-TISH tool [3]. By default, QC plots are created seperately for 5′ end matching (left panel) and mismatching (right panel) protein-coding gene mapped reads. In each panel, top figure shows the histogram of read lengths. Below, for each read length between 19 and 32 nts, one can see, from left-to-right, the frequency of in&out of frame reads, start codon periodicity, stop codon periodicity and CDS mapping profile of reads with that specific length.

[5] Zhang, P., He, D., Xu, Y., Hou, J., Pan, B. F., Wang, Y., Liu, T., Davis, C. M., Ehli, E. A., Tan, L., Zhou, F., Hu, J., Yu, Y., Chen, X., Nguyen, T. M., Rosen, J. M., Hawke, D. H., Ji, Z. & Chen, Y. 2017. Genome-wide identification and differential analysis of translational initiation. Nat Commun, 8, 1749.

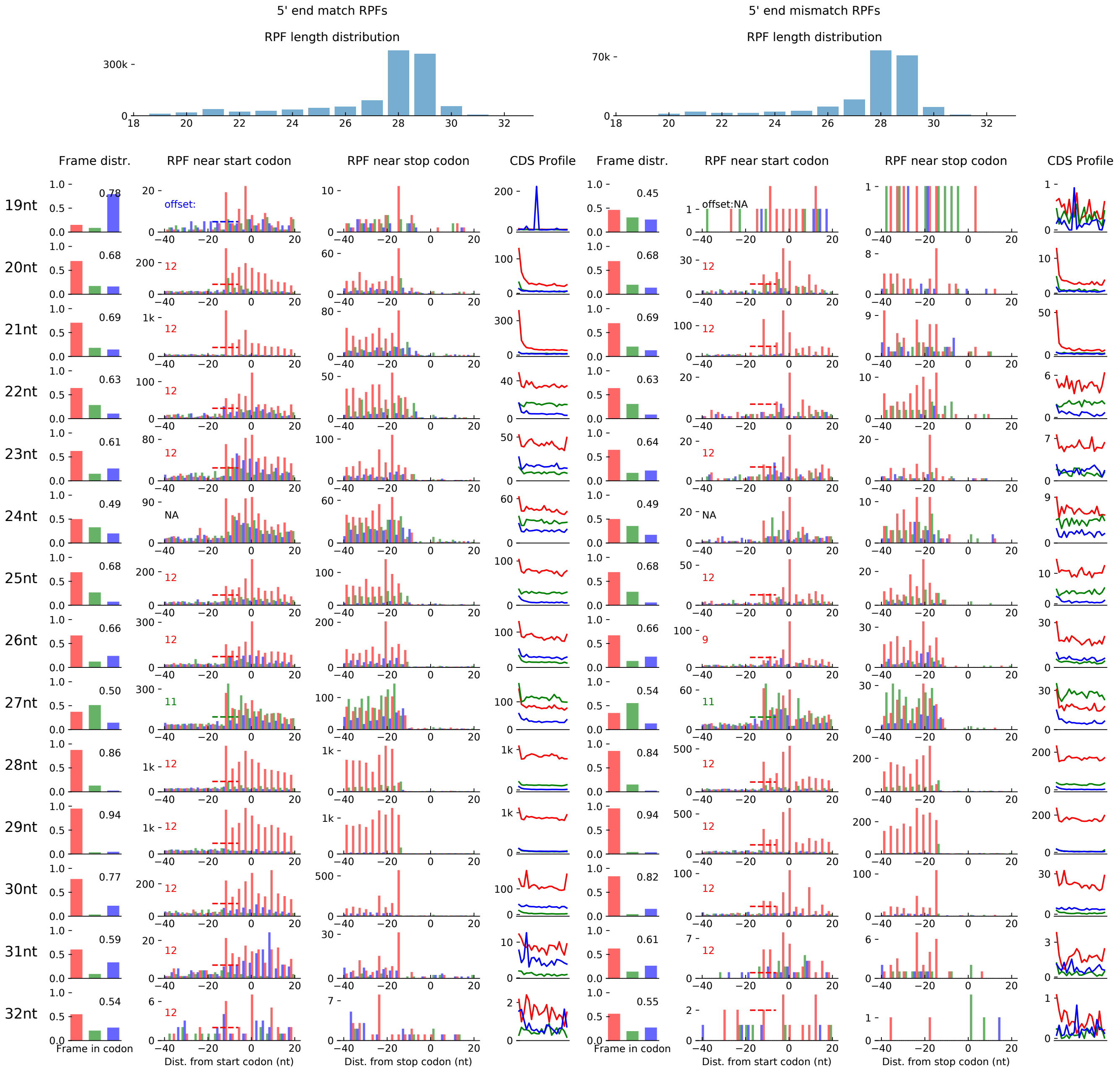

Supplementary Figure 32: Quality Control plots for the Ribo-seq experiment **org1_SET0**, generated by Ribo-TISH tool [3]. By default, QC plots are created seperately for 5′ end matching (left panel) and mismatching (right panel) protein-coding gene mapped reads. In each panel, top figure shows the histogram of read lengths. Below, for each read length between 19 and 32 nts, one can see, from left-to-right, the frequency of in&out of frame reads, start codon periodicity, stop codon periodicity and CDS mapping profile of reads with that specific length.

[5] Zhang, P., He, D., Xu, Y., Hou, J., Pan, B. F., Wang, Y., Liu, T., Davis, C. M., Ehli, E. A., Tan, L., Zhou, F., Hu, J., Yu, Y., Chen, X., Nguyen, T. M., Rosen, J. M., Hawke, D. H., Ji, Z. & Chen, Y. 2017. Genome-wide identification and differential analysis of translational initiation. Nat Commun, 8, 1749.

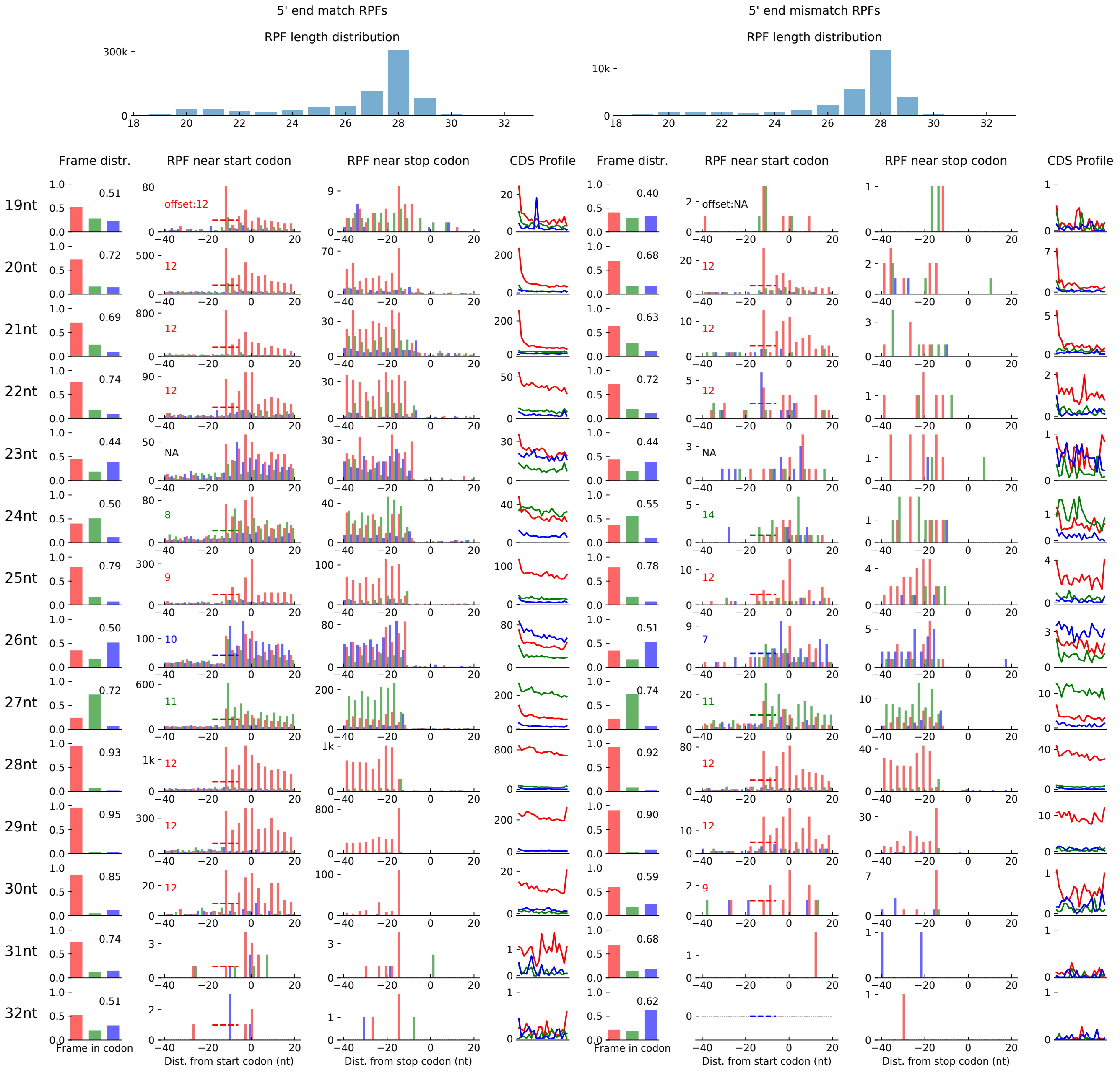

Supplementary Figure 33: Quality Control plots for the Ribo-seq experiment **org2_SET1**, generated by Ribo-TISH tool [3]. By default, QC plots are created seperately for 5′ end matching (left panel) and mismatching (right panel) protein-coding gene mapped reads. In each panel, top figure shows the histogram of read lengths. Below, for each read length between 19 and 32 nts, one can see, from left-to-right, the frequency of in&out of frame reads, start codon periodicity, stop codon periodicity and CDS mapping profile of reads with that specific length.

[5]Zhang, P., He, D., Xu, Y., Hou, J., Pan, B. F., Wang, Y., Liu, T., Davis, C. M., Ehli, E. A., Tan, L., Zhou, F., Hu, J., Yu, Y., Chen, X., Nguyen, T. M., Rosen, J. M., Hawke, D. H., Ji, Z. & Chen, Y. 2017. Genome-wide identification and differential analysis of translational initiation. Nat Commun, 8, 1749.

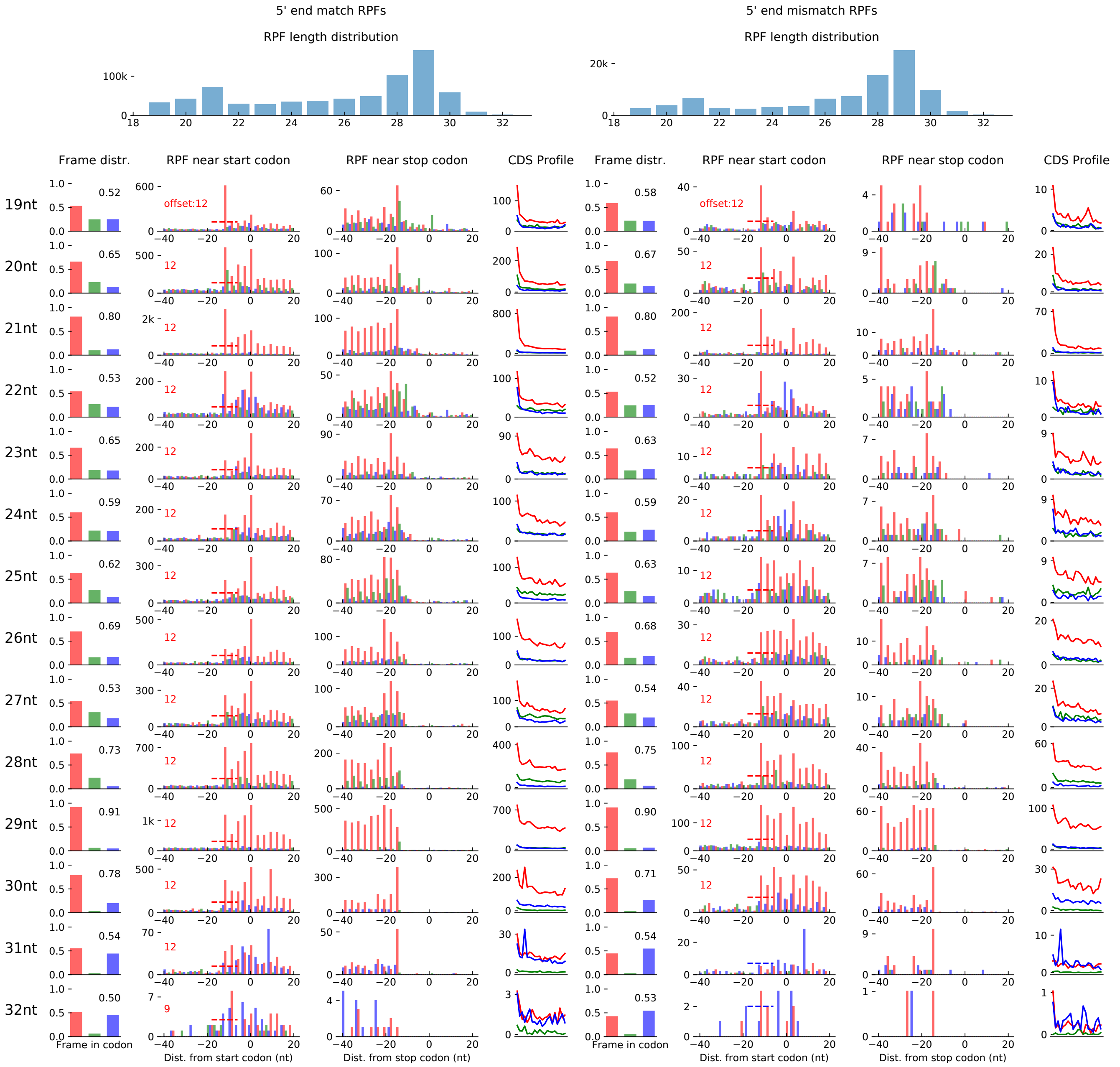

Supplementary Figure 34: Quality Control plots for the Ribo-seq experiment **org3_SET1**, generated by Ribo-TISH tool [3]. By default, QC plots are created seperately for 5′ end matching (left panel) and mismatching (right panel) protein-coding gene mapped reads. In each panel, top figure shows the histogram of read lengths. Below, for each read length between 19 and 32 nts, one can see, from left-to-right, the frequency of in&out of frame reads, start codon periodicity, stop codon periodicity and CDS mapping profile of reads with that specific length.

[5]Zhang, P., He, D., Xu, Y., Hou, J., Pan, B. F., Wang, Y., Liu, T., Davis, C. M., Ehli, E. A., Tan, L., Zhou, F., Hu, J., Yu, Y., Chen, X., Nguyen, T. M., Rosen, J. M., Hawke, D. H., Ji, Z. & Chen, Y. 2017. Genome-wide identification and differential analysis of translational initiation. Nat Commun, 8, 1749.
